# Multifunctional cerium nanolabels in electrochemical immunosensing with improved robustness and performance: determination of TIM-1 in colorectal cancer scenarios as a case study

**DOI:** 10.1007/s00604-025-07021-3

**Published:** 2025-03-19

**Authors:** Andrea Cabrero-Martín, Sara Santiago, Verónica Serafín, María Pedrero, Ana Montero-Calle, José M. Pingarrón, Rodrigo Barderas, Susana Campuzano

**Affiliations:** 1 Departamento de Química Analítica, Facultad de CC. Químicas, Universidad Complutense de Madrid, Pza. de Las Ciencias 2, 28040 Madrid, Spain; 2 Chronic Disease Programme, UFIEC, Institute of Health Carlos III, Majadahonda, 28220 Madrid, Spain; 3 CIBER of Frailty and Healthy Aging (CIBERFES), Instituto de Salud Carlos III, 28046 Madrid, Spain

**Keywords:** CeO_2_ multifunctional nanolabels, Nanozymes, Nanocarriers, Electrochemical immunoplatforms, TIM-1, Colorectal cancer

## Abstract

A multifunctional cerium oxide nanoparticles (CeO_2_NPs)-based nanolabel is exploited to implement an electrochemical sandwich-type immunoplatform for the determination of T-cell immunoglobulin and mucin domain 1 (TIM-1) biomarker, a mucin-like class I membrane glycoprotein associated with cancer angiogenesis. The immunoplatform is constructed using screen-printed electrodes where capture antibody is immobilized through the chemistry of diazonium salts. CeO_2_NPs exhibit robust pseudo-peroxidase activity even at high substrate concentrations. They are covalently functionalized in a simple manner after carboxylation with a detector antibody (dAb), acting dually as a nanozyme and nanocarrier for sensing bioreceptors. This allows the development of immunoplatforms with improved robustness and performance (in terms of a moderate enhancement in sensitivity, a significant expansion in the linear range, and a reduction in the background current) compared with the immunoplatforms prepared using nanolabels also decorated with the natural enzyme (horseradish peroxidase, HRP) or the conventional enzymatic labeling involving the dAb and an HRP-secondary antibody. Under the optimized experimental conditions, the developed electrochemical immunoplatform allows the highly sensitive detection of the TIM-1 glycoprotein, with a detection limit of 9.9 pg mL^−1^ and a linear working range of 33–600 pg mL^−1^. This performance permits biomarker quantification within clinically relevant ranges. This innovative configuration enables the precise diagnosis and stratification of colorectal cancer patients by analyzing plasma samples without pretreatment beyond a sample dilution and allows establishment of the first cut-off values reported for this purpose.

**Graphical abstract:**

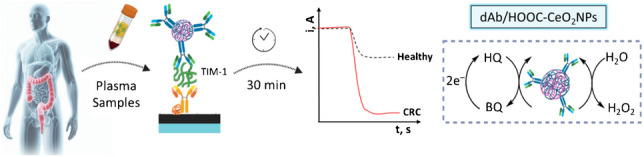

**Supplementary Information:**

The online version contains supplementary material available at 10.1007/s00604-025-07021-3.

## Introduction

In recent years, there has been an increasing interest in synthetic systems able to replicate biochemical reactions without relying on the complex structures of natural enzymes. These systems often involve nanomaterials with intrinsic enzyme-like properties and offer significant advantages by mimicking the catalytic behavior of enzymes while overcoming their limitations. By profiting from the robustness and versatility of nanomaterials with enzyme-like activity, these “nanozymes” are poised to have a great impact in fields such as diagnosis, prognosis and monitoring, therapeutics, and in environmental remediation [1–4].

Nanozymes combine inorganic nanomaterials, such as metal or metal oxide nanoparticles, with organic molecules or biomolecules to emulate the catalytic activity of natural enzymes [5], showcasing remarkable catalytic properties comparable to biological enzymes. Factors as size, shape, composition, crystal surface, charge, and hydrophilicity influence nanozymes activity levels [6]. In addition, nanozymes exhibit several advantages: (i) high stability; (ii) cost-effectiveness, while enzyme production processes are often complex and costly, inorganic nanomaterials can be produced with high efficiency and at a lower cost; (iii) recyclability, nanozymes maintain their catalytic activity across multiple cycles; (iv) multifunctionality, the huge surface area of nanozymes enables their coupling with multiple ligands fostering multifunctionality. The distinctive characteristics of these materials have propelled significant progress in biosensing, immunoassays, cancer diagnostics and therapy, neuroprotection, stem cell growth, and pollutant removal [7].

Recent advances have revealed amazing enzyme-like characteristics in nanomaterials such as derivatives of fullerenes, gold, rare earths, ferromagnetic nanoparticles [8], and cerium-based materials. These latter have received special attention in the past decade due to their superior redox features, high mechanical strength, good optical properties, thermal stability, and the behavior as nanozymes [9–11]. Indeed, CeO_2_ nanoparticles (CeO_2_NPs) have been exploited due to their remarkable pseudo-peroxidase activity which is intrinsically linked to their unique crystal structure and redox properties. These NPs adopt a fluorite-type structure, a robust and stable lattice that allows the coexistence of cerium in two oxidation states: Ce^3+^ and Ce^4+^. This dual-valence state facilitates the formation of catalytically active redox pairs, enhancing the efficiency of CeO_2_NPs as effective mimics of natural enzymes [12]. This behavior is of particular interest for the development of electrochemical biosensors, where their catalytic efficiency can significantly improve biomarker detection and signal generation. Moreover, CeO_2_NPs exhibit exceptional versatility for biochemical applications due to their ability to undergo diverse surface modifications. Among these modifications, carboxylation significantly enhances their interaction with biological molecules, such as antibodies. This interaction is achieved through stable covalent bonding with amine groups, which improves the stability of the biomolecules [13]. Taking advantage of the modification ability of CeO_2_NPs and their pseudo-peroxidase activity, this work reports their use as multifunctional nanolabels in electrochemical immunosensing. It is important to mention that most of the previous works used these NPs as electrode surface modifiers in label-free strategies [14–16]. Only a few works reported their use as nanolabels in sandwich-type configurations, but always combining CeO_2_NPs with other materials (Ag@CeO_2_ [17], Co_3_O_4_@CeO_2_-Au@Pt [18]) or even with the natural horseradish peroxidase (HRP) enzyme (CeO_2_NPs/MWCNTs-(HRP-Strept)) [19]. These strategies implied more complex preparation and did not take full advantage of the opportunities CeO_2_NPs offer as substitutes for natural enzymes.

Considering this background, this work reports the design, development, and characterization of a competitive electrochemical immunoplatform that leverages the multifunctionality of nanolabels formed with CeO_2_NPs, functioning as nanozymes and as detection bioreceptors nanocarriers, for the determination of the T-cell immunoglobulin and mucin domain 1 (TIM-1). TIM-1 is a mucin-like class I integral membrane glycoprotein [20] considered as an emerging clinical biomarker associated with aggressiveness in oncological processes and cancer angiogenesis [S1–S5], and other prevalent diseases [S6–S9]. Recent studies have elucidated the capability of TIM-1 to influence the tumor microenvironment. Correlative analyses reveal that its expression levels are in accordance with the presence of aggressive tumors, as heightened expression fosters cell migration and invasion while concurrently inhibits cell apoptosis. Furthermore, recent findings also suggest that the presence of this glycoprotein is associated with enhanced antitumor responses [12], so that the development of targeted simple, low-cost, and robust bioplatforms able to allow the high detectability and selectivity required for the determination of TIM-1 in real-world clinical scenarios is highly demanded. Due to their unique features, the use of multifunctional CeO_2_NP-based nanolabels is considered a good option for implementing robust and affordable electroanalytical immunosensing methods with the required practical performance.

Methods such as enzyme-linked immunosorbent assays (ELISAs) [21], lateral flow immunochromatography [22], and nuclear magnetic resonance (NMR) [23] have been reported for the determination of TIM-1. However, these methods are often time-consuming, expensive, and require complex sample preparation and specialized equipment. Some electrochemical immunosensors for TIM-1 have been already reported [20, 24–27]. However, even though they provide adequate analytical characteristics, they have not been applied in real clinical scenarios. The immunoplatform developed and characterized in this work involves the use of screen-printed carbon electrodes (SPCEs) modified by electrografting with *p*-aminobenzoic acid (*p*-ABA) to efficiently immobilize the TIM-1 specific capture biotinylated antibody (btn-cAb) through covalently attached neutravidin (Neu). A sandwich-format immunoassay was thereafter implemented by means of detection antibody (dAb)-decorated CeO_2_NPs as multifunctional nanolabels that provide pseudo-peroxidase activity to amplify the electrochemical response. The results obtained demonstrate the role played by these CeO_2_NPs-based multifunctional nanolabels in the electrochemical immunosensing of TIM-1 improving moderately or significantly some key characteristics (robustness, sensitivity, linear range, and background current) and allowing the analysis of samples in a relevant clinical setting (plasma samples from patients with colorectal cancer at different stages).

## Experimental

### Reagents, solutions, and samples

All commercial solvents and reagents used for the synthesis of dAb/HOOC/CeO_2_NPs were used as received, without further purification. Purified water was produced by a Millipore Milli-Q purification system (18.2 MΩ cm). Hydrochloric acid (HCl, 35%) was procured from Merck. Cerium nitrate (Ce(NO_3_)_3_ × 6H_2_O, 99%), ammonium hydroxide (NH_4_OH, 35%), sulfuric acid (H_2_SO_4_, 63–65%), and nitric acid (HNO_3_, 65%) were obtained from Sigma-Aldrich. Sodium hydroxide (NaOH) and ethanol (EtOH, 95%) were supplied by Scharlab. 2-(*N*-morpholino)ethanesulfonic acid (MES, ≥ 99.0%) was sourced from GERBU Biotechnik GmbH. Disodium hydrogen phosphate (Na_2_HPO_4_), sodium chloride (NaCl, ≥ 99.0%), potassium chloride (KCl), and sodium dihydrogen phosphate dihydrate (NaH_2_PO_4_ × 2H_2_O) were acquired from Scharlab.

Additional reagents were of the highest analytical grade available. *N-*(3-dimethylaminopropyl)-*N′*-ethylcarbodiimide (EDC) was obtained from Across Organics, while *N-*hydroxysulfosuccinimide (sulfo-NHS) was provided by Fluorochem. Hydroquinone (HQ), horseradish peroxidase (HRP), casein, Tween® 20, hydrogen peroxide (H_2_O_2_, 30% w/v), potassium ferricyanide trihydrate (K_3_Fe(CN)_6_ × 3H_2_O), potassium ferrocyanide trihydrate (K_4_Fe(CN)_6_ × 3H_2_O), HRP-conjugated anti-mouse IgG (RABHRP2), bovine serum albumin (BSA), biotin (btn), and 2,2′-azino-bis-(3-ethylbenzothiazoline)−6-sulfonic acid (ABTS) were purchased from Sigma-Aldrich. 3,3′,5,5′-tetramethylbenzidine (TMB) and neutravidin (Neu) were sourced from Thermo Fisher Scientific. Sodium nitrite (NaNO_2_) was obtained from Panreac, and 4-aminobenzoic acid (*p-*ABA) from Across.

All buffer solutions were prepared using purified water. A blocker casein solution (Ref. 37528, casein blocking buffer, BB solution), which was a ready-to-use phosphate buffer solution containing 1% w/v purified casein, and Pierce™ Protein-Free PBS (Ref. 37572, protein-free blocking buffer, PFBB solution) were purchased from Thermo Fisher Scientific. Phosphate-buffered saline (PBS) solutions of 10 mM at pH 7.4, containing 137 mM NaCl and 2.7 mM KCl, 0.05 M phosphate buffer solution at pH 6.0 (PB), 0.025 M MES buffer at pH 5.0, and 0.1 M sodium phosphate buffer composed of PBS with an addition of 0.05% Tween® 20 (PBST) at pH 7.4, were also prepared.

Additional solutions include a mixture of EDC and sulfo-NHS (each at 50 mg mL^−1^) prepared in 0.025 M MES buffer at pH 5.0 for the activation of HOOC-CeO_2_NPs. For electrochemical measurements, stock solutions of 0.1 M HQ and 0.1 M hydrogen peroxide (H_2_O_2_) were prepared in 0.05 M PB just before use.

Biotinylated goat anti-human TIM-1 capture antibody (btn-cAb), mouse anti-human TIM-1 detection antibody (dAb), and the recombinant human TIM-1 standard were acquired as the components of the human TIM-1/KIM-1/HAVCR DuoSet ELISA kit purchased from R&D Systems. For the selectivity studies, potential non-target interfering human proteins were evaluated, including hemoglobin (Hb), serum albumin (HSA, ≥ 96%), and serum immunoglobulin G (hIgG), all sourced from Sigma-Aldrich. Additionally, recombinant tumor necrosis factor-alpha (TNF, ≥ 96%), obtained from BD Bioscience and recombinant interleukin-13 receptor alpha-2 (IL-13Rα2, from Human IL-13Rα2 DuoSet ELISA) and recombinant periostin protein (POSTN, from Human Periostin/OSF-2 DuoSet ELISA) procured from R&D Systems, were tested.

### Apparatus and electrodes

Amperometric measurements were carried out at room temperature using a CHI812B potentiostat (CH Instruments, Inc.) controlled by the CHI812B software. Cyclic voltammetry (CV) and electrochemical impedance spectroscopy (EIS) measurements were carried out using a FRA2 µAutolab Type III potentiostat/galvanostat (Metrohm Autolab B.V., The Netherlands) controlled by the GPES & FRA software (Eco Chemie B.V., The Netherlands).

Screen-printed carbon electrodes (SPCEs, DRP-110) with a 4-mm diameter carbon working electrode were sourced from Metrohm-DropSens. These electrodes included a carbon counter electrode and an Ag pseudo-reference electrode. The specific cable connector (DRP-CAC) that provided the interface between the SPCEs and the potentiostat was also obtained from Metrohm-DropSens. The amperometric measurements were taken under constant stirring at 350 rpm using 10mL glass Pobel electrochemical cells. Additional equipment employed included a Crison Basic 20 + pH meter, a P-Selecta Ultrasons ultrasonic bath, a Heidolph Reax Top homogenizer for small samples, a Meditronic P-Selecta oven, a Velp scientifica magnetic stirrer hot plate, a BA04 Sartorius Secura analytical balance, and an MPW-65R centrifuge from MPW (Med. Instruments).

The synthesized materials were characterized using different techniques with the following equipment: Ultraviolet–visible (UV–Vis) measurements were performed using an HP 8453 UV–Vis diode spectrophotometer controlled by HP Chemstation software; attenuated total reflectance Fourier transform infrared spectroscopy (ATR-FTIR) was conducted with a Shimadzu FTIR-8300 spectrophotometer and controlled by the OPUS V 5.1 software; transmission electron microscopy (TEM) images were acquired with a JEM 2100 PLUS microscope operating at 200 kV; and X-ray diffraction (XRD) characterization was carried out using a LynxEye X-ray diffractometer, with Cu Kα radiation (*λ* = 1.54060 Å), in a Bragg-Bretano configuration.

### Preparation of dAb/HOOC-CeO_2_NPs

The synthesis of CeΟ_2_NPs was conducted using a precipitation method previously reported in the literature, with minor modifications [S10]. In this procedure, 50 mL of a 0.2 M Ce(NO_3_)_3_ × 6H_2_O solution was mixed with 25 mL of a 3 M NH_4_OH solution under constant stirring at room temperature overnight. A precipitate was formed gradually changing in color from white to gray. The resulting suspension was washed three times with deionized water by centrifugation at 14,000 rpm for 5 min per cycle. The creamy white solid obtained was subsequently dried in an oven at 60 °C for 12 h.

The carboxylation of CeΟ_2_NPs was carried out through a partial oxidation reaction in a (3:1) H_2_SO_4_/HNO_3_ medium following the procedure described by Centane et al. [13]. Specifically, 1 g of the synthesized CeΟ_2_NPs was incubated in 5 mL of the oxidizing mixture with gentle stirring at room temperature for 8 h. The resulting carboxyl-functionalized nanoparticles (HOOC-CeΟ_2_NPs) were washed with a H_2_O/EtOH mixture (3:1 ratio) until neutral pH and centrifuged at 14,000 rpm for 5 min. Finally, the nanoparticles were dried in an oven at 60 °C for 24 h.

dAb/HOOC-CeO_2_NPs were prepared by using a protocol analogous to that previously reported for the fabrication of nanocarriers involving multi-walled carbon nanotube (MWCNT) nanohybrids [S11, S12]. A 0.2 mg mL^−1^ aqueous suspension of HOOC-CeO_2_NPs was prepared in purified water. The supernatant was removed, and the resulting product was incubated in the dark, under continuous stirring at room temperature for 3 h, with 200 μL of a solution containing 0.4 M EDC and 0.1 M sulfo-NHS in 10 mM PBS, pH 7.4. The activated HOOC-CeO_2_NPs were conjugated by incubation for 24 h at 4 °C under continuous stirring in a 0.5 μg mL^−1^ of dAb solution in PBS. The dAb/HOOC-CeO_2_NPs nanolabels were centrifuged at 14,000 rpm for 5 min, washed three times with PBS, and finally resuspended in 500 μL of 100 mM PBST, pH 7.4. The nanocarriers were stored at 4 °C under continuous stirring until use.

### Preparation of the immunoplatform

The functionalization of the SPCEs working electrodes via reductive electrochemical grafting with *p*-ABA was made according to a previously reported protocol [S13]. Initially, diazonium ion (HOOC-Phe-N_2_^+^) was synthesized by gradually adding a 2 mM NaNO_2_ aqueous solution to a 3 mM *p*-ABA solution in 1 M HCl, maintaining the mixture in an ice bath (38 µL NaNO_2_ for 2 mg of *p-*ABA). The reaction was carried out under stirring for 7 min. To covalently bind the diazonium ion to the SPCE surface, each electrode was immersed in the diazonium solution, and ten consecutive voltammetric cycles were applied in the 0.0 to − 1.0 V potential range (*ν* = 0.2 V s^−1^ vs. the Ag pseudo-reference electrode). Finally, the modified SPCEs were thoroughly rinsed with purified water and allowed drying at room temperature.

The carboxyl groups grafted on the working electrode surface (*p*-ABA/SPCE) were activated by depositing 10 μL of an EDC/sulfo-NHS (100 mM each) solution freshly prepared in 25 mM MES buffer of pH 5.0, allowing the reaction to proceed for 30 min. After washing with the same MES buffer, 5 µL of a 900µg mL^−1^ Neu solution prepared in MES buffer was added and incubation let to proceed for 45 min. After washing with MES buffer, 5 µL of a 5µg mL^−1^ btn-cAb solution prepared in 100 mM PBS pH 7.4 was added followed by a 10min incubation time.

After rinsing with PBS, minimization of nonspecific interactions was carried out by blocking with 10 μL of 1% (w/v) casein allowing incubation for 30 min and followed by a washing step with PBS. The affinity reaction with TIM-1 was accomplished by adding 5 µL of TIM-1 standard solution (or the samples to analyze) prepared in BB solution onto the working electrode and incubating for 30 min. After washing with PBS, 5 µL of dAb/HOOC-CeO_2_NPs nanolabels was dropped on the modified working electrode surface and incubated for 30 min. The immunosensing platform was rinsed again with 10 mM PBS at pH 7.4 and maintained with a 25μL drop of the same buffer until the electrochemical measurements were carried out. All incubation steps during the immunoplatform assembly were conducted at room temperature in a humid environment to avoid drop evaporation.

### Electrochemical measurements

The modified electrode was immersed in a measurement cell containing 10 mL of 50 mM PB at pH 6.0 and 50 μL of a freshly prepared 100 mM HQ solution. The amperometric measurements were carried out under constant stirring and applying − 0.2 V against an Ag pseudo-reference electrode. After the background current was stabilized (~ 20 s), 50 μL of a freshly prepared 100 mM H_2_O_2_ solution in 50 mM PB at pH 6.0 was added [S14]. Due to the pseudo-peroxidase activity of CeO_2_NPs, a cathodic reduction current of H_2_O_2_ mediated by HQ was recorded. Due to the immunoassay format used, the increase in the cathodic current was directly proportional to the TIM-1 concentration. The amperometric responses correspond to the difference between the steady-state current (which was reproducibly recorded in less than 100 s avoiding bias in the results) and the background current, obtained after and before the addition of H_2_O_2_, respectively. In the manuscript, the results are expressed in terms of the Δ*i* values, which correspond to the difference between the amperometric signals measured with immunoplatforms prepared in the presence and in the absence of btn-cAb for each antigen concentration. In addition, unless otherwise specified, they were the mean values of three replicates, and the error bars displayed were estimated as three times the standard deviation of each set of three replicates (*α* = 0.05).

The nanolabels and the stepwise fabrication of the immunosensor were characterized by cyclic voltammetry (CV) and electrochemical impedance spectroscopy (EIS) using a 5 mM [Fe(CN)_6_]^3‒/4‒^ solution in 0.1 M KCl. In CV, current intensity variations were scanned over a potential range from − 0.3 to + 0.7 V (vs. the Ag pseudo-reference electrode) at a scan rate of 50 mV s^−1^. EIS measurements were performed under open-circuit conditions, with a sinusoidal excitation amplitude of 10 mV (RMS) applied over a frequency range from 0.04 to 1 × 10^5^ Hz. The automatic analyzer was set to achieve a 0.001% standard deviation (S.D.) for the *I*(*jω*) correlator output, with a cut-off time of 100 s. Data were collected with 10 points per decade within the specified frequency range.

### Evaluation of pseudo-peroxidase activity

To evaluate the pseudo-peroxidase catalytic activity, 1 mg of each compared nanomaterial was placed in a 1.5-mL centrifuge tube. Subsequently, 250 μL of a TMB/H_2_O_2_ solution was added, and the development of a blue color was assessed after 2 min of reaction.

To quantify the enzymatic activity (in units per milligram of solid), the standard protocol provided by Sigma-Aldrich was followed [S15]. For this purpose, a 5 mg mL^−1^ ABTS solution was prepared in 0.025 M MES buffer at pH 5.0. The reaction mixture consisted of 40 μL of the suspension of each nanomaterial (1 mg mL^−1^) and 40 μL of a 0.3% w/w H_2_O_2_ solution. The absorbance at 405 nm was monitored using a UV–Visible spectrophotometer, with measurements recorded every 20 s over a 3-min period. A blank control was prepared without nanomaterial, following the same procedure.

### Analysis of human plasma samples

This study for the biomarker validation was approved by the Ethical Review Board of the Instituto de Salud Carlos III (CEI PI 13_2020-v2). Plasma samples from both healthy individuals (CT) and colorectal cancer (CRC) patients were sourced after the approval of the Ethical Committee from the biobank of Hospital Clínico San Carlos. Written informed consent was obtained from all participants enrolled in the study. The samples were stored at – 80 °C until utilized, adhering strictly to ethical guidelines and regulations governing sample handling and experimental protocols.

Following confirmation of the absence of matrix effects in 1/2 diluted plasma samples, the determinations were performed by simple interpolation of the amperometric responses obtained with the immunoplatforms into the calibration plot constructed with external standard solutions. Furthermore, ROC (receiver operating characteristic) curves were constructed using R software (version 3.6.2), and employing the “ModelGood” and “Epi” packages to assess diagnostic performance.

## Results and discussion

**Fig. 1:**
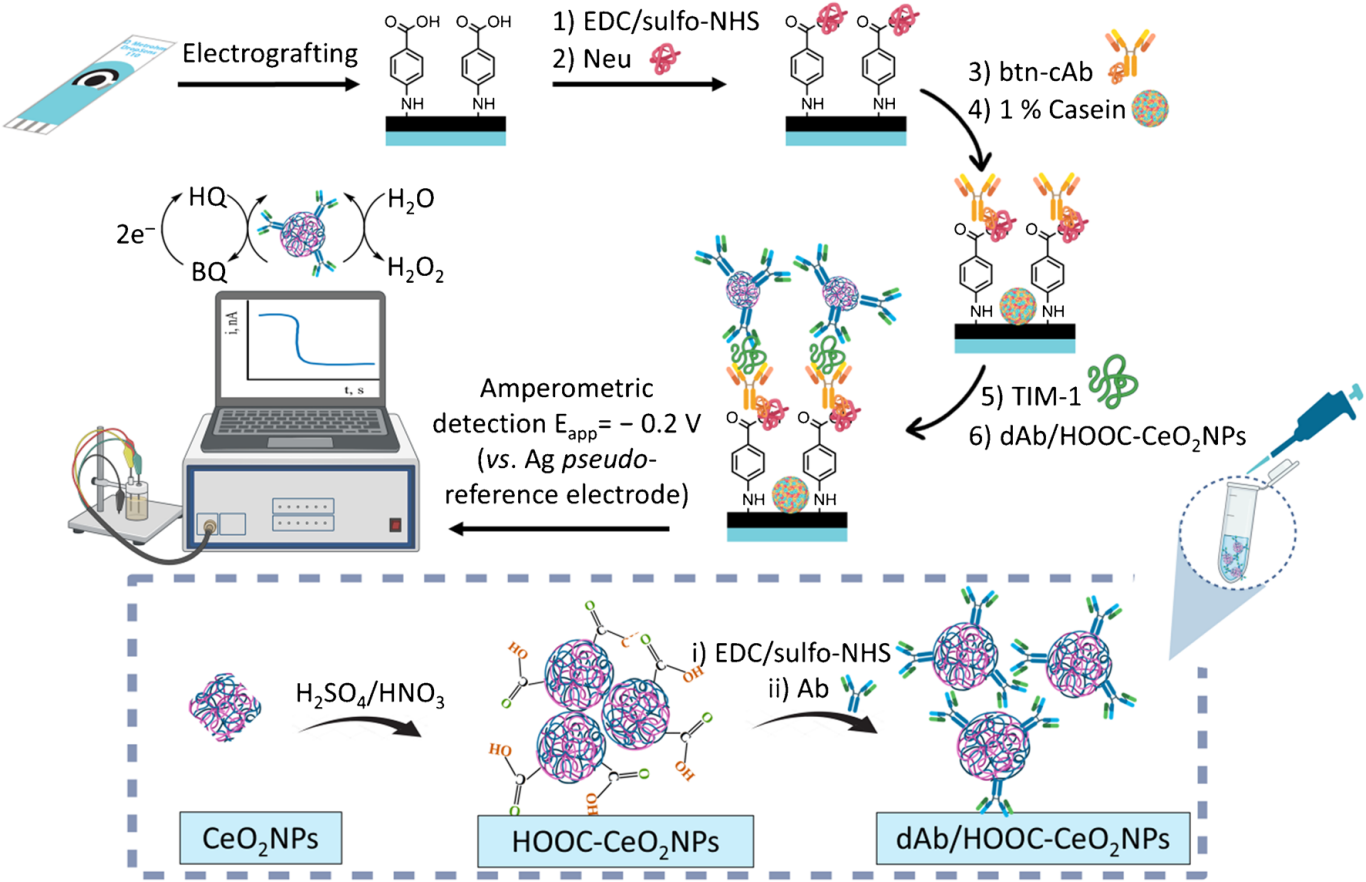
Schematic illustration of the sequential steps involved in the preparation of dAb/HOOC-CeO_2_ NPs nanolabels and the construction of the sandwich immunoplatform for the determination of TIM-1 using amperometric transduction

The immunoplatform developed in this work was constructed on SPCEs modified by *p*-ABA electrografting, followed by covalent attachment of Neu to further immobilize the specific btn-cAb through the affinity reaction between biotin and Neu. After the target capturing, a sandwich assay configuration was implemented by incorporation of the multifunctional dAb/HOOC-CeO_2_NPs nanolabels. TIM-1 detection was carried out by monitoring changes in the cathodic current using amperometry in the presence of H_2_O_2_ and hydroquinone (HQ). This method leveraged the pseudo-peroxidase activity of CeO_2_NPs, able to catalyze the H_2_O_2_ reduction with HQ serving as redox mediator at a potential of ‒ 0.2 V (vs. Ag pseudo-reference electrode), producing a cathodic current directly proportional to the concentration of TIM-1. Figure 1 outlines the sequential steps for the preparation of the dAb/HOOC-CeO_2_NPs nanocarriers, the construction of the immunoplatform, and the amperometric detection.

### Characterization of the nanolabels

The different prepared nanolabels (CeO_2_NPs, HOOC-CeO_2_NPs, and dAb/HOOC-CeO_2_NPs) were exhaustively characterized using morphological, structural, elemental, and electrochemical techniques.

**Fig. 2:**
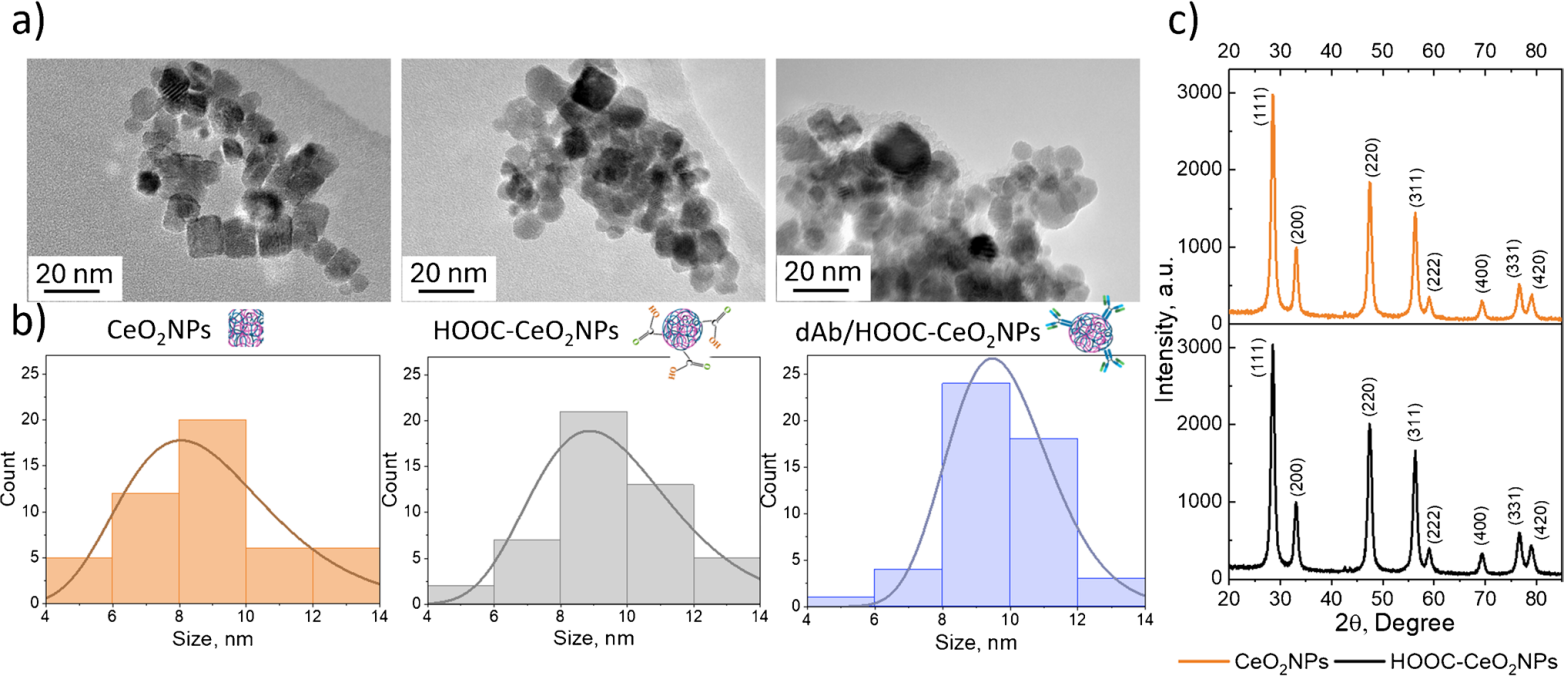
**a** CeO_2_NP, HOOC-CeO_2_NPs, and dAb/HOOC-CeO_2_NPs TEM micrographs. **b** Histograms of NPs diameter distribution obtained from TEM for *n* = 50 (Gaussian fitting of the particle size distribution is overlapped in dark line on the histogram bars). **c** Diffraction patterns for CeO_2_NPs and HOOC-CeO_2_NPs

Their shape, size, and spatial distribution were analyzed using TEM (Fig. 2a). Morphologically, unmodified CeO_2_NPs exhibited well-defined cubic structures, consistent with that reported in literature [28], while HOOC-CeO_2_NPs and dAb/HOOC-CeO_2_NPs tended to adopt a spherical shape.

Unmodified CeO_2_NPs exhibited higher dispersion compared to HOOC-CeO_2_NPs and dAb/HOOC-CeO_2_NPs, which showed a more pronounced degree of aggregation. This indicates successful surface functionalization with carboxyl groups and dAb, as hydrogen bonding interactions between these groups likely promote nanoparticle aggregation [29]. The dAb/HOOC-CeO_2_NPs displayed a peripheral halo, which should correspond to the presence of the dAb, proving their successful functionalization.

The high resolution of the TEM images allowed for the visualization of lattice fringes, confirming the crystalline nature of the NPs in all cases. Size histograms (*n* = 50) (Fig. 2b) showed a narrow size distribution, reflecting uniform synthesis conditions. The average sizes were found to be (8.9 ± 0.6) nm for CeO_2_NPs, (9.6 ± 0.5) nm for HOOC-CeO_2_NPs, and (9.8 ± 0.3) nm for dAb/HOOC-CeO_2_NPs.

The crystalline structure of the nanomaterials was further characterized using XRD (Fig. 2c). The XRD pattern of untreated CeO_2_NPs revealed eight peaks corresponding to the crystal planes (111), (200), (220), (311), (222), (400), (331), and (420), indicative of a fluorite structure [30, 31]. The XRD pattern of HOOC-CeO_2_NPs showed similar results, confirming that the crystal structure remained largely unchanged during chemical modification of the NP surface, consistent with the TEM observations and previous studies [13].

The FTIR spectra of CeO_2_NPs and HOOC-CeO_2_NPs are displayed in Fig. S1a (Supporting Information). For CeO_2_NPs, two prominent bands at 1429 cm^−1^ and 1310 cm^−1^ were observed that correspond to the stretching vibration mode of the O-Ce–O bond [S16]. The presence of COOH groups on the surface of HOOC-CeO_2_NPs was confirmed by a carbonyl stretching vibration at 1686 cm^−1^, accompanied by additional bands at 1450, 1282, 1094, and 1022 cm^−1^. The bands at 1282 and 1094 cm^−1^ were associated with the O-Ce–O stretching mode, while those at 1450 and 1022 cm^−1^ were attributed to overlapping Ce-OH vibrations [S17]. Additionally, a band around 2916 cm^−1^ was ascribed to C-H stretching, followed by a broad band corresponding to the -OH groups in the carboxylic moieties [13].

The materials used were further characterized by UV–Vis spectroscopy. As illustrated in Fig. S1b (Supporting Information), CeO_2_NPs display an absorption band around 300 nm [S18], corresponding to charge transfers from oxygen 2p to cerium 4f orbitals, confirming the formation of CeO_2_ [S19]. The HOOC-CeO_2_NPs spectrum is like that of CeO_2_NPs, with a slight shift in the absorption bands attributable to resonance effects induced by COOH groups [13].

**Fig. 3:**
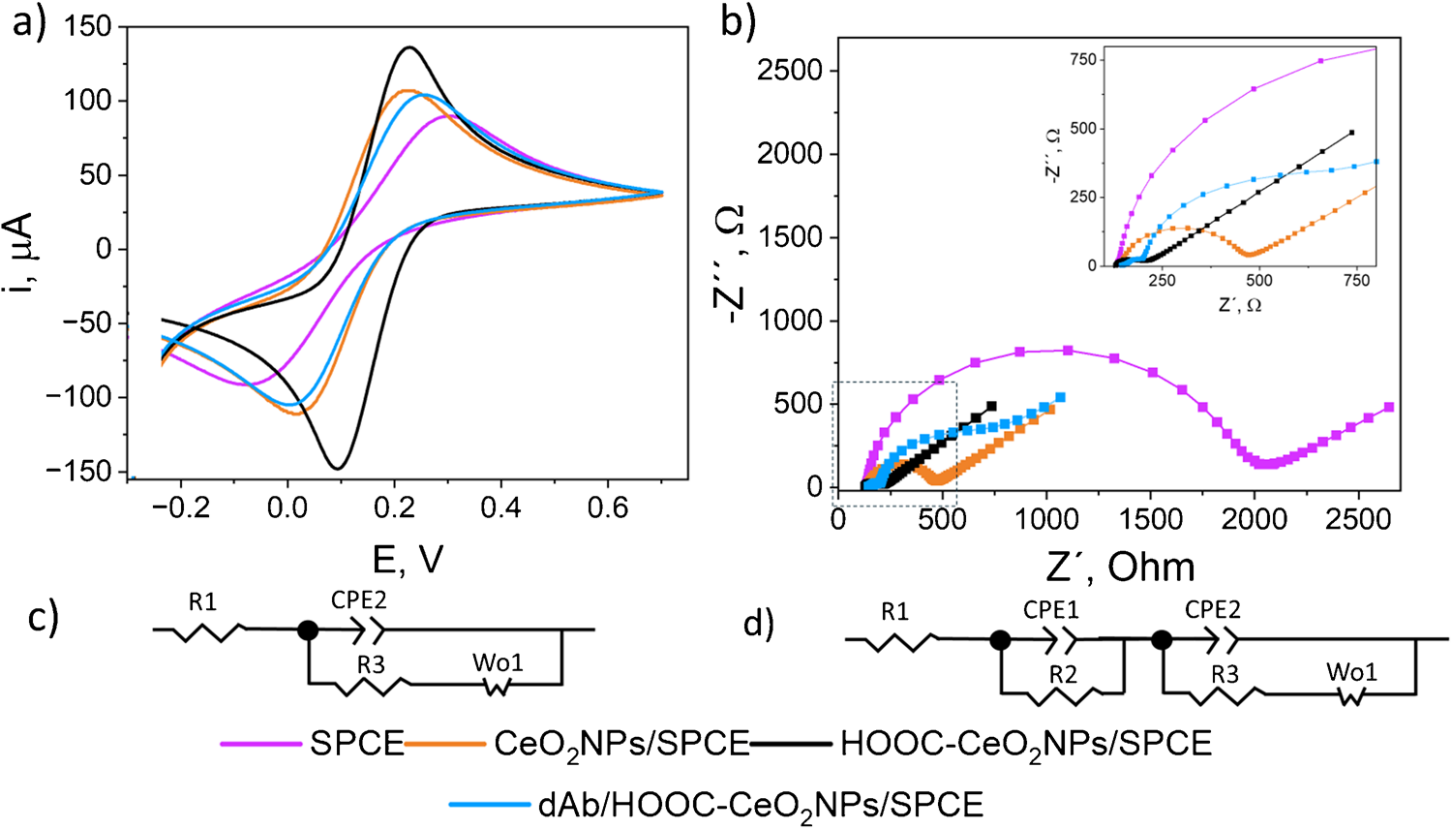
**a** Cyclic voltammograms (scan rate, 50 mV s^−1^) and **b** Nyquist spectra (scanning frequencies: 10^5^ – 0.04 Hz; amplitude = 0.01 V, applied potential: 0 V) for the [Fe(CN)_6_]^3‒/4‒^ redox pair. Electrodes: SPCE (purple), CeO_2_NPs/SPCE (orange), HOOC-CeO_2_NPs/SPCE (black), and dAb/HOOC-CeO_2_NPs/SPCE (blue). Equivalent circuits used to fit the experimental data where R1 is the bulk resistance (Rs), and R2 and R3 are the charge transfer resistances (*R*_CT_), CPE is the constant phase element, and *W* is the Warburg element corresponding to SPCE, CeO_2_NPs/SPCE and HOOC-CeO_2_NPs/SPCE (**c**), and dAb/HOOC-CeO_2_NPs/SPCE

The electrochemical characterization of the nanolabels was carried out by drop casting modification of the SPCEs working electrode with 5 μL of 1 mg mL^−1^ suspensions of each nanomaterial and allowing drying. Subsequently, CVs and EIS responses were recorded using a 5 mM [Fe(CN)_6_]^3‒/4‒^ in 0.1 M KCl solutions as redox probe (Fig. 3a and b, respectively). The impedance spectra were presented as Nyquist plots and fitted with the appropriate equivalent circuit (Fig. 3c and d).

As shown in Fig. 3a, the CeO_2_NPs/SPCE exhibited more reversible behavior and higher current intensity peaks compared to the unmodified electrode (SPCE), indicating that CeO_2_NPs enhanced the charge transfer rate. Modification with HOOC-CeO_2_NPs resulted in improved current intensities, which was attributed to the enhanced ability of cerium atoms to alternate between Ce^3+^ and Ce^4+^ upon carboxylation, generating oxygen vacancies in the fluorite lattice structure and enhancing electrostatic interactions with anionic electroactive species such as the [Fe(CN)_6_]^3‒/4‒^ probe [12, 32, 33]. These observations were further confirmed by performing voltammograms using Ru^3+^/Ru^4+^ as redox probe where an opposite trend was observed upon comparison of CeO_2_NPs/SPCE and HOOC-CeO_2_NPs (data not shown). The proper immobilization of the dAb on the HOOC-CeO_2_NPs was evidenced by a decrease in the anodic and cathodic peak current intensities reflecting the insulating nature of the antibody and the subsequent decrease of the electron transfer rate at the electrode surface.

Impedance spectra confirmed these observations (Fig. 3b). The charge transfer resistance values (*R*_CT_) were obtained by fitting the experimental data to the Randles equivalent circuit illustrated in Fig. 3c (SPCE, CeO_2_NPs/SPCE, and HOOC-CeO_2_NPs/SPCE) and Fig. 3d (dAb/HOOC-CeO_2_NPs/SPCE). As can be seen, the diameter of the semicircle decreased upon modification of the SPCE with CeO_2_NPs, indicating a reduction in the *R*_CT_ attributed to the semiconducting properties of cerium nanoparticles (*R*_CT, SPCE_ = 1800 Ω; *R*_CT, CeO2NPs_ = 46 Ω). Electrodes modified with HOOC-CeO_2_NPs displayed an even smaller semicircle diameter, due to the enhanced electrostatic interactions after carboxylation discussed above (*R*_CT, HOOC-CeO2NPs_ = 28 Ω). Modification with dAb/HOOC-CeO_2_NPs resulted in two semicircles, indicating the coexistence of two charge transfer processes: a smaller semicircle associated with rapid electron transfer influenced by HOOC-CeO_2_NPs (*R*_CT 1, dAb/HOOC-CeO2NPs_ = 30 Ω) and a larger semicircle suggesting a slower charge transfer due to antibody modification (*R*_CT 2, dAb/HOOC-CeO2NPs_ = 49 Ω).

### Evaluation of the pseudo-peroxidase catalytic activity and kinetic studies of the nanolabels

**Fig. 4:**
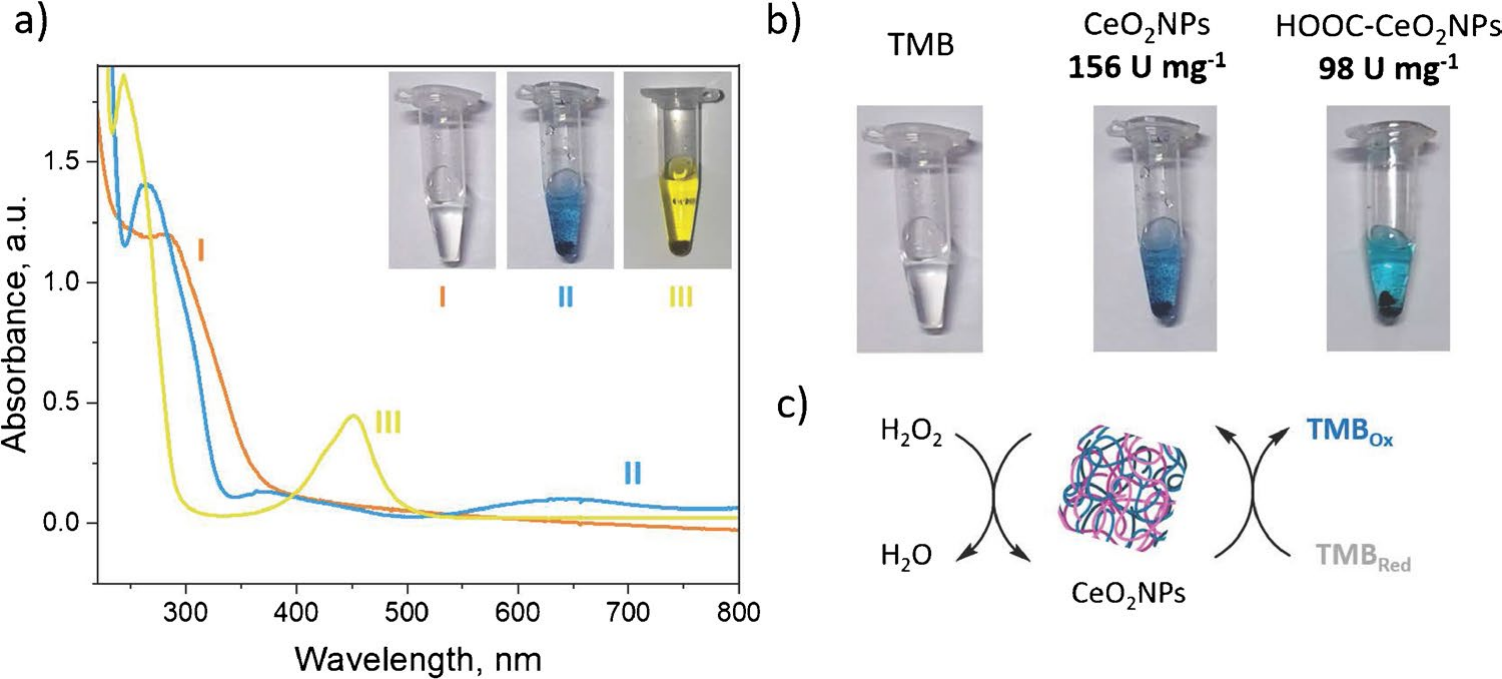
**a** UV–Vis spectra corresponding to: (I) CeO_2_NPs (orange), (II) CeO_2_NPs + TMB/H_2_O_2_ (blue), and (III) after the addition of H_2_SO_4_ (yellow). **b** Color change observed for TMB/H_2_SO_2_ upon adding either CeO_2_NPs or HOOC-CeO_2_NPs. **c** Pseudo-enzymatic cascade reaction mediated by CeO_2_NPs or HOOC-CeO_2_NPs nanozymes in the presence of H_2_O_2_, leading to the oxidation of TMB and a consequent color change from colorless to blue

CeO_2_NPs pseudo-peroxidase activity was evaluated via TMB oxidation in the presence of H_2_O_2_ [9]. As illustrated in Fig. 4a-I, CeO_2_NPs exhibited an absorption band at 300 nm. Upon the addition of 100 μL of a commercial TMB/H_2_O_2_ reaction mixture, a new absorption band appeared at 650 nm, corresponding to the blue coloration of the solution (Fig. 4a-II) [S18]. This indicated the formation of the TMB radical cation, demonstrating the pseudo-peroxidase activity of CeO_2_NPs. Subsequently, to halt the reaction, 50 μL of 1 M H_2_SO_4_ was added, which led to the disappearance of the absorption band at 650 nm and the appearance of a new band at 450 nm, observing a solution color change from blue to yellow, which was attributed to the formation of a diimine species (Fig. 4a-III).

The pseudo-enzymatic activity was quantified, in units per milligram of nanozyme/enzyme, using ABTS and following the Sigma-Aldrich protocol equation [S15]. CeO_2_NPs pseudo-enzymatic activity was determined to be 156 U mg^−1^, while that for HOOC-CeO_2_NPs resulted to be 98 U mg^‒1^. These results agreed with the color intensity variations observed (Fig. 4b) during the TMB/H_2_O_2_ reaction (Fig. 4c) and confirmed that the NP pseudo-peroxidase activity increased with surface area [9] and that TMB oxidation was triggered by a Fenton-like reaction induced by CeO_2_ and not naturally. Although the synthesized nanozymes exhibited a slightly lower catalytic activity compared to natural HRP (219 U mg^‒1^), this activity was sufficient for developing electrochemical immunoplatforms attractive in terms of reduced cost and improved robustness as it is demonstrated in the following sections.

Amperometric measurements at − 0.2 V vs. the pseudo-reference Ag electrode, under constant stirring, were utilized to study the pseudo-peroxidase activity kinetics for CeO_2_NPs and HOOC-CeO_2_NPs. The variation in the current intensity observed after the addition of increasing concentrations of H_2_O_2_, ranging from 1.5 to 6.0 mM, into a cell containing 0.5 mM HQ in 50 mM phosphate buffer (PB) at pH 6.0 was used to determine the *K*_app_ corresponding to the pseudo-peroxidase activity of the synthesized nanozymes.

Firstly, the obtained results were fitted to the Hill equation (Eq. 1), after being linearized according to Eq. 2 where [*S*] was the substrate concentration (H_2_O_2_), *i* was the current intensity variation, *i*_max_ was the background current intensity at the steady state, and ${K}_{M}^{\text{app}}$ was the apparent Michaelis–Menten constant. The parameter “*x*,” known as the Hill coefficient, measures the cooperativity of substrate binding to the protein. In this case, when plotting log[(*i*_max_/*i*) − 1] vs*.* log[H_2_O_2_], a linear relationship was observed with a slope value close to – 1 in all cases (*x* = (1.1 ± 0.2) for CeO_2_NPs, as an example), which reduced Eq. 1 to the Michaelis–Menten form. This indicated the absence of substrate binding cooperativity, suggesting either a single (or multiple) binding site in the, herein, nanozyme that did not interact cooperatively. This confirmed that the synthesized nanozymes showed a good fit to the Michaelis–Menten model [S20, S21].

1 $$\frac{i}{{i}_{\text{max}}}= \frac{{[S]}^{x}}{{K}_{M}^{\text{app}}+ {[S]}^{x}}$$

2 $$\text{log}\left[\left(\frac{{i}_{\text{max}}}{i}-1\right)\right]=\text{log}{K}_{M}^{\text{app}}-x\text{log}\left[S\right]$$

On the other hand, nanozymes generally exhibit a higher resistance to saturation compared to natural enzymes such as HRP. This distinction arises from the fact that nanozymes function through heterogeneous catalysis, with their activity governed by surface interactions and the availability of active sites. In contrast, HRP can experience saturation at high substrate concentrations due to its reliance on Michaelis–Menten kinetics. Nanozymes, on the other hand, offer a greater density of active sites distributed across their surface. Furthermore, their catalytic performance is not constrained by structural limitations or substrate binding affinities that regulate natural enzymes [S22], as evidenced by the results shown in Fig. S2 (Supporting Information), where the amperometric responses obtained for high H_2_O_2_ concentrations using HRP-modified SPCEs and dAb/HOOC-CeO_2_NPs in the presence of HQ were compared.

Nevertheless, at low substrate concentrations, enzymatic reactions following the Michaelis–Menten model exhibit first-order kinetics. Therefore, by plotting the neperian logarithm of the current intensity over time using the amperometric responses from 1.5 mM substrate solutions (not shown), a regression line was obtained whose slope provided the apparent kinetic constant, *K*_app_. The comparison of the *K*_app_ values calculated for the prepared nanozymes and commercial HRP are provided in Table S1 (Supporting Information).

These results, consistent with those of the spectrophotometric characterization (Fig. 4), showed that CeO_2_NPs exhibited the most favorable kinetics among all the NPs tested, although lower than that of the natural enzyme. Moreover, the stepwise functionalization of CeO_2_NPs with carboxyl (-COOH) groups and dAb molecules did not significantly impair their pseudo-enzymatic kinetics. All these results suggested the potential of the synthesized nanomaterials as artificial enzymes to develop sensitive and robust immunoplatforms.

### Optimization of key experimental variables involved in the immunoplatform preparation and functioning

All experimental variables related to the fabrication and amperometric response of the immunoplatform were systematically optimized. The criterion for selecting the variables was the maximum ratio obtained from the currents measured in the presence of 500 pg mL^−1^ TIM-1 (T signal) versus those measured in the absence of the target (B signal). The following variables were tested: concentration and incubation time of Neu; concentration and incubation time of btn-cAb; type, concentration, and incubation time of the blocking agent; incubation time for TIM-1; HOOC-CeO_2_NPs loading; dAb loading in the dAb/HOOC-CeO_2_NPs; incubation time with the dAb/HOOC-CeO_2_NPs; and catalytic system used in the electrochemical readout. The results of these optimization studies are provided in Fig. S3 (Supporting Information) and summarized in Table S2 (Supporting Information).

The impact of Neu concentration was evaluated by comparing the amperometric responses of different immunoplatforms prepared utilizing Neu concentrations ranging from 0 to 2000 μg mL^−1^. As shown in Fig. S3a, a larger T/B ratio was achieved for a Neu concentration of 900 μg mL^−1^. Therefore, this concentration was chosen for further work. Regarding the incubation time for Neu binding, a 45-min incubation period was selected, as it provided a better T/B ratio with longer times resulting in a decrease in the specific signal (Fig. S3b) which may be attributed to poorer recognition of TIM-1 since many btn-cAb molecules can be immobilized due to the large Neu loading on the surface. The electrochemical responses of immunoplatforms prepared by varying btn-cAb concentrations (from 0 to 10 μg mL^−1^) (Fig. S3c) showed as high concentrations resulted in lower T/B ratios due to the decrease in the T signals due to impaired target recognition when a lot of btn-cAb was immobilized. Consequently, 5 μg mL^−1^ was selected as the btn-cAb concentration for subsequent experiments. Regarding the btn-cAb incubation time, a 10min period was found to be sufficient for the affinity interaction between Neu and btn (Fig. S3d). Different blocking agents, commonly used to minimize nonspecific adsorption of immunoreagents on modified electrodes, were tested. As shown in Fig. S3e, a larger T/B ratio was obtained using 2% casein in PBS buffer. Further optimization of casein concentration and incubation time revealed that 1% casein provided better results (Fig. S3f) with a 30-min effective incubation time (Fig. S3g). Larger amounts of casein resulted in a decrease of the specific signals without a significant decrease of the non-specific adsorptions. The target protein incubation time (Fig. S3h) gave rise to a slight increase for the specific signals up to 30 min, then decreasing slightly together with the T/B ratio. Thus, a 30-min incubation period was selected for the effective binding of TIM-1 onto the casein/btn-cAb/Neu/*p*-ABA-SPCEs. The influence of the HOOC-CeO_2_NPs loading was evaluated over the 0 to 1 mg mL^−1^ range. As shown in Fig. S3i, the T/B ratio increased with the HOOC-CeO_2_NPs loading, reaching a higher value for 0.2 mg mL^−1^. Larger loadings provided leveled off non-specific signals while T decreased probably due to nanoparticle aggregation preventing binding of the dAb. The effect of the dAb solution concentration used to decorate the HOOC-CeO_2_NPs yielded a larger T/B ratio for 0.5 μg mL^−1^, which was therefore selected (Fig. S3j). The incubation time for the dAb/HOOC-CeO_2_NPs bioconjugate was checked over the 15 to 90 min range. As shown in Fig. S3k, the intensity of specific signals decreased after 30 min, likely due to a more impeded recognition of the target or to possible aggregation phenomena. Furthermore, since we were working with a pseudo-enzyme, the effect of the substrate and redox mediator concentrations and the potential to be applied for amperometric detection were evaluated. The effect of H_2_O_2_ concentration was checked over the 0.25 to 5 mM range, with the results depicted in Fig. S3l. As expected, the specific current increased rapidly with the H_2_O_2_ concentration, with the dAb/HOOC-CeO_2_NPs showing an exceptional stability even at high H_2_O_2_ concentrations. This remarkable stability was attributed to the ability of CeO_2_NPs to toggle between Ce^3+^ and Ce^4+^ oxidation states, thus endowing them with a notable advantage in terms of regenerating their active state, thereby potentially enhancing their catalytic efficiency in contrast to the natural HRP which is prone to denaturation for high H_2_O_2_ concentrations (as shown by the results in Fig. S2) [S23]. These results highlighted the enhanced robustness of synthetic nanozymes compared to their natural enzyme counterparts. Considering the T/B ratios, the selected H_2_O_2_ concentration was 1 mM. Moreover, Fig. S3m shows as the T/B ratio increased with the HQ concentration up to 1 mM, then decreasing for larger concentrations which is likely due to electrode fouling.

Figure S4a shows the CV behavior of the HQ system in the presence of H_2_O_2_, at the immunosensor. When compared with the voltammogram obtained at the bare SPCE, a splitting of peaks was observed. This can be attributed to a mechanistic change caused by the interaction between HQ and the antibody, as reported in the literature [34]. The second reduction peak appeared at around − 0.15 V, and therefore a slightly more negative potential was necessary to fully reduce the electroactive species. At potential values close to − 0.3 V, an increase in the faradaic currents was seen due to the background reduction. This behavior was corroborated by performing amperometric measurements at different potentials (Fig. S4b), showing that the background current in the absence of TIM-1 increased for potential values more negative than − 0.3 V. Although a larger T/B ratio was observed at − 0.3 V, considering the minimal differences observed between − 0.2 and − 0.3 V, and as a compromise between sensitivity and selectivity, − 0.2 V was selected as the potential to be applied for amperometric detection.

### Stepwise electrochemical characterization of the immunoplatform preparation

The immunoplatform preparation, using the experimental conditions selected above, was monitored step by step by CV and EIS, using 5 mM Fe(CN)_6_^4-^/^3-^ as redox probe. The corresponding Nyquist plots are shown in Fig. S5a and S5b (Supporting Information). As observed, the modification of the SPCE with *p*-ABA provoked a significant increase of the charge transfer resistance (*R*_CT, SPCE_ 1800 Ω; *R*_CT, *p*-ABA/SPCE_ 11000 Ω), which was attributed to the electrostatic repulsion between the anionic redox probe and the − COO^-^ groups present on the electrode surface under the measurement pH conditions (Fig. S5a). The subsequent activation with EDC/sulfo-NHS led to a reduction in the *R*_CT_ value due to the neutralization of the − COO^-^ groups (*R*_CT EDC/sulfo-NHS/*p*-ABA/SPCE_ 5200 Ω). The *R*_CT_ value increased again after the immobilization of Neu (*R*_CT, Neu/*p*-ABA/SPCE_ 15000 Ω), due to the formation of a protein layer that inhibited charge transfer on the electrode surface. The subsequent immobilization of the btn-cAb resulted in a decrease of the electron transfer resistance *R*_CT, btn-cAb/Neu/*p*-ABA/SPCE_ 10000 Ω), possibly due to a reorganization of the Neu layer induced by its interaction with the btn-cAb [S24]. Incubation with 1% casein led to a drastic decrease in *R*_CT_ (Fig. S5b)_._ It is important to mention that, from this step, the impedance spectra showed two different semicircles corresponding to an electrode surface on which two layers are deposited during the immunoplatform preparation. A similar behavior was reported previously for other immunosensors involving incubation with casein [S11, S25]. The equivalent circuit *R*_1_(*R*_2_*Q*_1_)(*Q*_2_[*R*_3_*W*_1_]) displayed in Fig. S5d corresponded to an electrode surface coated with two layers and best fitted the experimental results (*R*_CT, casein/btn-cAb/Neu/*p*-ABA/SPCE_ 490 Ω + 2600 Ω). The incorporation of the target protein TIM-1 caused an increase in the second semicircle area in the Nyquist plot, consistent with the formation of a layer that made charge transfer more difficult (*R*_CT, TIM-1/casein/btn-cAb/Neu/*p*-ABA/SPCE_ 420 Ω + 2800 Ω). Finally, the addition of the nanocarriers (dAb/HOOC-CeO_2_NPs) resulted in a decrease in *R*_CT_ (*R*_CT, dAb/HOOC-CeO2NPs/TIM-1/casein/btn-cAb/Neu/*p*-ABA/SPCE_ 290 Ω + 2500 Ω), attributed to the conductive nature of CeO_2_NPs, which facilitated electron transfer. The observed behavior aligned with the results observed by CV (Fig. S5c and S5d).

### Analytical operational characteristics and selectivity

**Fig. 5:**
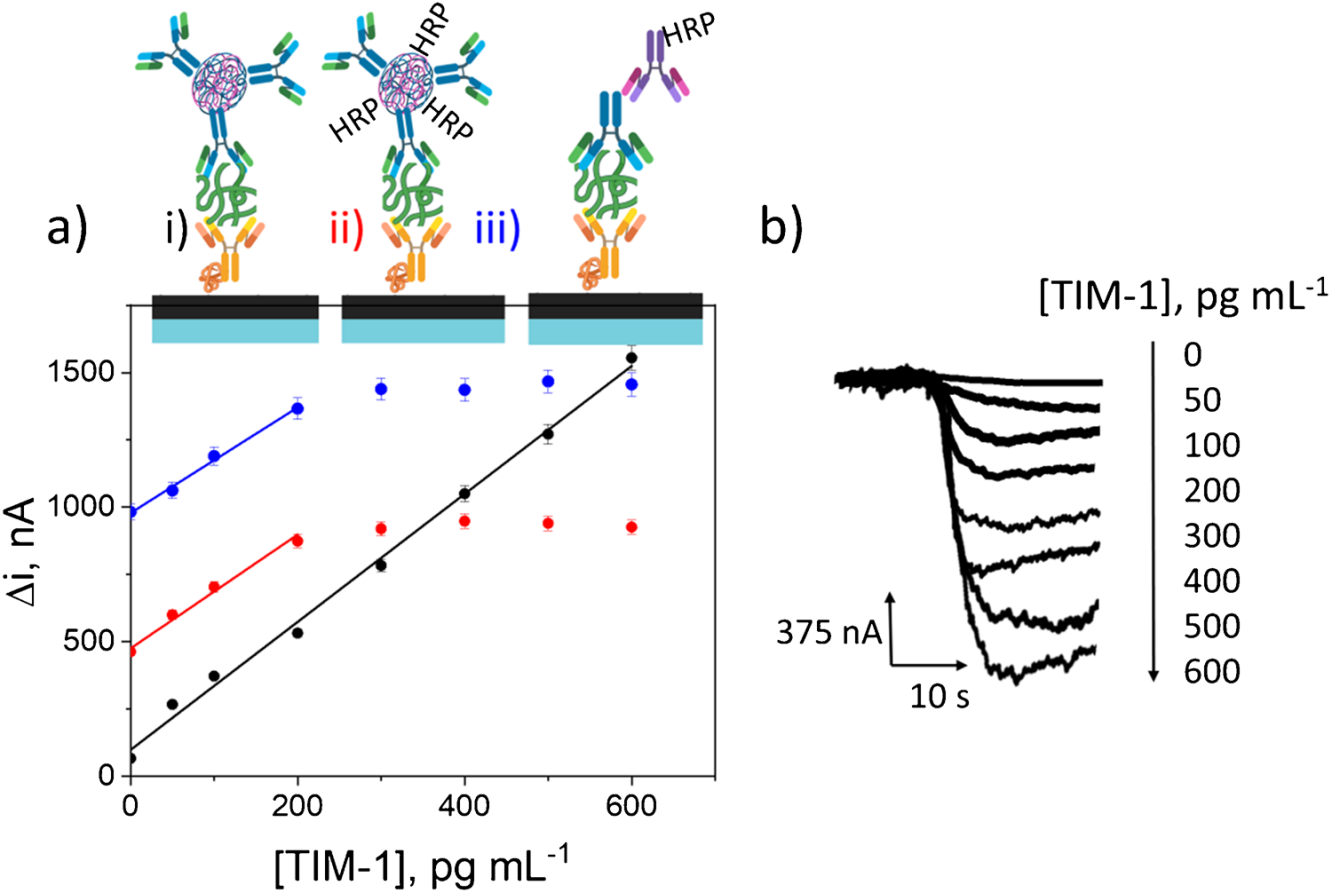
**a** Calibration graphs for the determination of TIM-1 constructed with immunoplatforms involving the use of dAb/HOOC-CeO_2_NPs (black line), dAb + HRP/HOOC-CeO_2_NPs (red graph), and HRP-anti-mIgG/dAb (blue graph) for the recognition and enzymatic labeling of the target TIM-1. **b** Amperometric signals recorded with the dAb/HOOC-CeO_2_NPs-based immunoplatform for different TIM-1 concentrations

The analytical and operational characteristics of the immunoplatform developed for the determination of TIM-1 were evaluated. To check the advantages of the used nanolabel, the calibration plots constructed with immunoplatforms prepared using nanolabels decorated with and without HRP (dAb + HRP/HOOC-CeO_2_NPs and dAb/HOOC-CeO_2_NPs) as well as using the conventional enzymatic labeling involving the dAb and an HRP-secondary antibody (HRP-anti-mIgG) were compared. The respective calibration graphs are displayed in Fig. 5. The synthesized HOOC-CeO_2_NPs nanocarriers without HRP improved the sensitivity by a 12% and widened the linear concentration range compared to that provided using dAb + HRP/HOOC-CeO_2_NPs. The use of dAb/HOOC-CeO_2_NPs (black line in Fig. 5) allowed a strong linear correlation between the amperometric response and TIM-1 concentration in the 33–600 pg mL^−1^ concentration range. The corresponding fitting equation was − *i* (*nA*) = (2.38 ± 0.06) [TIM-1, pg mL^−1^] + (98 ± 22), with an excellent correlation coefficient of *r*^2^ = 0.995. However, when the dAb + HRP/HOOC-CeO_2_NPs nanomaterial was employed (red line), a shorter linear concentration range, up to 200 pg mL^−1^, and a smaller slope value were apparent, − *i* (*nA*) = (2.1 ± 0.2) [TIM-1, pg mL^−1^] + (475 ± 16), with a correlation coefficient of *r*^2^ = 0.990. These results showed clearly that the dAb/HOOC-CeO_2_NPs nanolabel provided sufficient pseudo-peroxidase activity and did not require decoration with HRP to provide immunosensing with the required sensitivity. It is worth noting also the significantly higher intercept value and the shorter linear range obtained using dAb + HRP/HOOC-CeO_2_NPs nanolabels. The latter can be attributed to the lower number of dAb molecules on the NPs when HRP molecules were also incorporated.

The comparison with the conventional strategy using recognition and labeling with HRP-anti-mIgG/dAb also showed a slightly higher sensitivity ((2.38 ± 0.06) vs. (2.0 ± 0.1) nA mL pg^−1^), a broader linear range, and an intercept almost 10 times lower (98 vs 978 nA) using the multifunctional nanolabel (dAb/HOOC-CeO_2_NPs), highlighting the advantages it offers in electrochemical immunosensing.

The limit of detection (LOD = 3 × s_B_/slope) and limit of quantification (LOQ = 10 × s_B_/slope) values for the dAb/HOOC-CeO_2_NPs-based immunoplatform were estimated to be 9.9 and 33 pg mL^−1^, respectively (*s*_*B*_, standard deviation of the blank, *n* = 10).

A study on TIM-1 levels in plasma from patients with clear-cell carcinoma established a threshold of 142 pg mL^−1^ [35]. Therefore, it can be concluded that the LOD and LOQ values achieved with the developed immunoplatform allow the clinical differentiation between healthy individuals and cancer patients.

Table 1 compares the analytical performance and main characteristics of the developed immunoplatform with those reported with other electrochemical immunoassays and immunosensors for TIM-1. In terms of analytical performance, the LOD achieved was comparable to some reported values [20], although higher than others [24, 26], but still within the clinically relevant range mentioned above. It is also important to highlight here that none of the reported electroanalytical methods for the determination of TIM-1 were applied in real scenarios.

**Table 1:** Analytical performance and key features of electrochemical immunoassays/immunosensors reported for the determination of TIM-1

Detection strategy	Technique	Linear range, pg mL^−1^/LOD, pg mL^−1^	Assay preparation time/test time	Storage stability	Sample	Reference
Sandwich immunoassay with cAb on AuNPs-modified GCE, and dAb-decorated ABEI-PEI-PFO dots-RGOs/PtNPs as nanolabels	ECL	0.0500–1000/0.0167	cAb/AuNPs/GCE ~ 13 h; ABEI-PEI-PFO dots-RGOs/Pt NP@Ab_2_-BSA^e)^: ~ 18 h/not reported	Not reported	Spiked serum	[24]
Sandwich immunoassay with specific cAb on CuCl_2_ NWs and AuNP-modified GCE, and dAb-decorated PdPtBP MNPs/MXene as nanolabels	DPV	500–100000/86	cAbs/CuCl_2_ NWs/AuNPs/GCE ~ 2 h; dAb/PdPtBP MNPs/MXene: ~ 49 h; CuCl_2_ NWs: ~ 10 min/ ~ 2 h	cAbs/CuCl_2_ NWs/AuNPs/GCE, and PdPtBP MNPs/MXe nanolabels: 10 days	Spiked urine	[25]
Sandwich immunoassay with specific cAbs on COF-AuNPs-modified GCE, and dAb-decorated NiCo_2_S_4_@CeO_2_ microspheres as nanolabels	DPV	0.010–50/0.002	cAbs/COFs/AuNPs/GCE ~ 1 h 10 min; COFs-AuNPs: ~ 1 h 15 min; Ab_2_/NiCo_2_S_4_@CeO_2_: ~ 43 h/ ~ 15 min	cAbs/COFs/AuNPs/GCE: 7 weeks; dAb-NiCo_2_ S_4_ @CeO_2_ nanolabels: not reported	Spiked plasma	[26]
Label-free immunosensor with specific cAbs on Au-Galinstan Nd-modified disposable paper electrode	EIS	100–1000000/64	cAbs/Au-Galinstan Nds/paper electrode > 1 h; Au-Galinstan Nds: ~ 72 h/not reported	cAbs/Au-Galinstan Nds/paper electrode: 20 days	Spiked serum	[27]
Sandwich immunoassay with cAb-MBs and dAbs labeled with Strep-HRP and detection at SPCEs	Amperometry	87–7500/26	cAb-MBs: 2.2 h/45 min	cAb-MBs: 22 days	Cultured cancer cell extracts	[20]
Sandwich immunosensor based on the use of btn-cAb/Neu/*p*-ABA/SPCE and dAb/HOOC-CeO_2_NPs as nanolabels	Amperometry	33–600/9.9	Assay preparation time: casein/btn-cAb/Neu/*p*-ABA/SPCEs: 2 h; dAb/HOOC-CeO_2_NPs: 3.5 days (HOOC-CeO_2_NPs: 56 h and 27 h for dAb attachment)/test time: 60 min	btn-cAb/Neu/pABA/SPCE: 30 days; dAb/HOOC-CeO_2_NP nanolabels: 7 days	Plasma from CRC patients	This work

*ABEI*, aminobutylethyl isoluminol; *Ab*_*2*_, secondary antibody; *Au-Galinstan Nds*, gold-Galinstan nanodroplets; *AuNPs*, gold nanoparticles; *BSA*, bovine serum albumin; *cAb*, capture antibody; *COFs*, covalent organic frameworks; *CRC*, colorectal cancer; *NWs*, nanowires; *dAb*, detection antibody; *DPV*, differential pulse voltammetry; *ECL*, electrochemiluminescence; *EIS*, electrochemical impedance spectroscopy; *GCE*, glassy carbon electrode; *Strep-HRP*, streptavidin horseradish peroxidase; *MBs*, magnetic beads; *MNPs*, magnetic nanoparticles; *MXene*, two-dimensional transition metal carbides/nitrides; *PdPtBP*, palladium-platinum bipyramids; *PEI*, polyethyleneimine; *PFO*, perfluorooctanoate; *PtNPs*, platinum nanoparticles; *RGOs*, reduced graphene oxides; *SPCEs*, screen-printed carbon electrodes

Interestingly, the developed immunoplatform also exhibited a slightly better LOD compared to the commercial ELISA kit using the same immunoreagents (9.9 pg mL^−1^ vs. 15.6 pg mL^−1^ ELISA Kit). Additionally, it avoided the need for expensive enzymes, offered a significantly faster detection time (60 min vs. 4 h 30 min starting from the casein/btn-cAb/Neu/*p*-ABA-SPCEs and cAb-coated ELISA plate, respectively), and can be used with portable, cost-effective instrumentation, making it ideal for decentralized settings and in field applications.

The reproducibility in the preparation of the multifunctional nanolabel was checked by determining its pseudo-catalytic activity using three different batches. An average value of 156 U mg^−1^ with a relative standard deviation (RSD) of 3.1% was obtained. In addition, the reproducibility of the amperometric measurements made with the developed immunoplatform was evaluated by comparing the amperometric responses for 200 pg mL^−1^ TIM-1 obtained with different immunoplatforms prepared in the same manner using the same or different nanolabel batches. The RSD values ​obtained, 2.9% (*n* = 8) and 3.6% (*n* = 7), respectively, confirmed the reproducibility of the protocols for the preparation of the nanolabel and the immunoplatform as well as for the amperometric transduction, ensuring the robust performance of the immunoplatform even when different batches of dAb/HOOC-CeO_2_NPs were used.

The storage stability of the immunoplatforms and the dAb/HOOC-CeO_2_NPs nanolabels once prepared was evaluated separately. This evaluation was carried out by comparing the amperometric responses obtained over time in the presence and absence of the TIM-1 standard (500 pg mL^−1^) (Fig. S6, Supporting Information). As it can be seen, the prepared immunoplatforms provided stable responses for at least 30 days while the stability of the bionanoconjugates was 7 days.

The selectivity of the developed immunoplatform was evaluated by testing various non-target interfering proteins that might be present in biological samples. The tested interfering compounds included circulating proteins (hIgG, Hb, and HSA) as well as circulating and tumor tissue biomarkers (TNF, POSTN, and IL-13Rα2) [36–41]. These potential interferents were checked at the concentrations commonly found in biological samples. The cross-reactivity of each non-target protein was examined both in the presence (500 pg mL^−1^) and in the absence of TIM-1. The results shown in Fig. S7 (Supporting Information) indicated that there were no significant differences in the T/B ratios in the presence of any of the tested proteins.

### Analysis of plasma samples from CRC patients

The developed immunoplatform was used to analyze 16 plasma samples from healthy (H) individuals (*n* = 8) and CRC patients (*n* = 8), diagnosed at various stages of the disease (I–IV). Three replicates were performed for each sample, and the mean and associated error were calculated.

To evaluate the potential presence of a matrix effect, the slope value of the calibration curve constructed with TIM-1 standards ((2.38 ± 0.06) nA mL pg^−1^) was statistically compared with those constructed in twofold diluted plasma samples of a healthy individual ((2.4 ± 0.1) nA mL pg^−1^). Clearly, no significant differences between the slope values, with a *t*_exp_ value of 1.24, which is below the critical *t*-value of 2.776 (*n* = 3, *α* = 0.05), were found.

**Fig. 6:**
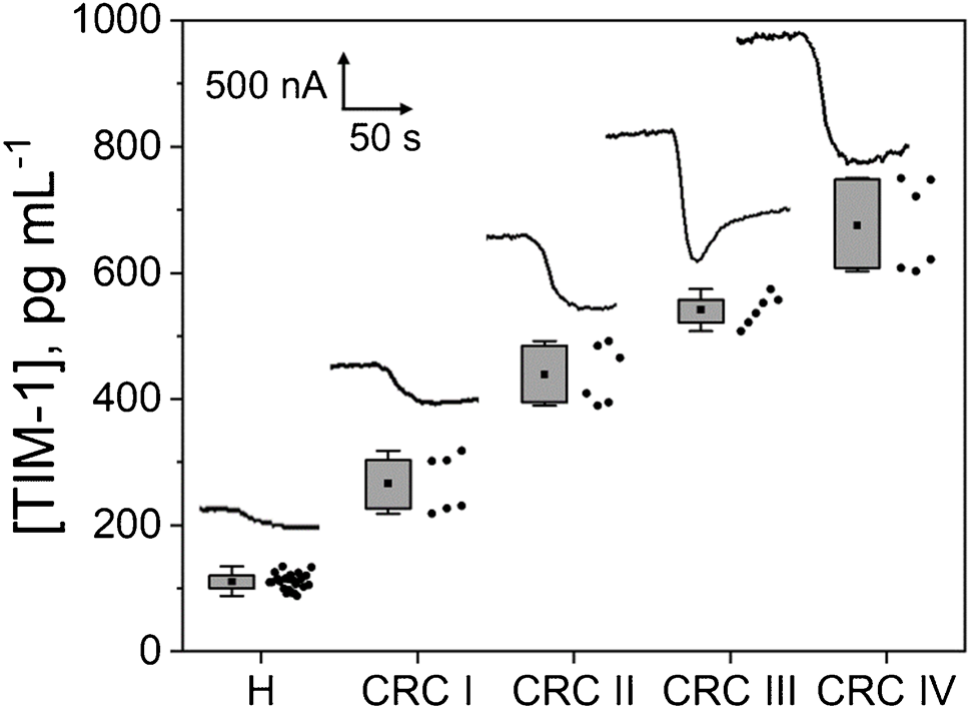
TIM-1 concentrations determined with the developed immunoplatform in plasma samples grouped into pools of H individuals and CRC patients according to their stage. A representative amperometric curve for each group is also shown

Therefore, as no apparent matrix effect was observed under these conditions, the TIM-1 concentration in the samples was determined by simple interpolation of the amperometric signals obtained for the 2-times diluted plasma samples into the calibration plot constructed with TIM-1 standards. The results, shown in Fig. 6, compare the concentrations found in H individuals with those obtained for CRC patients at different disease stages.

The results obtained for the determination of TIM-1 in this kind of sample suggested a significantly higher plasma concentration in CRC patients compared to H individuals, with an increase of TIM-1 levels in parallel to CRC progression. In this sense, CRC patients at more aggressive and metastatic stages (stages III and IV) presented higher TIM-1 plasma levels than CRC patients at non-metastatic stages of the disease (stages I and II). To evaluate the diagnostic potential of the developed bioanalytical platforms in CRC patients, and to determine the optimal cut-off values for reliable discrimination among different stages of the disease, ROC curve analysis was performed (Fig. S7, Supporting Information). These curves revealed that the cut-off value for discriminating CRC patients and H individuals was 176.45 pg mL^−1^ with 100% specificity and 100% sensitivity. This cut-off value is the first one reported for TIM-1 in CRC and is similar to that claimed for clear-cell carcinoma (142 pg mL^−1^), an uncommon salivary gland carcinoma [35].

Furthermore, the ROC curve analysis suggested the usefulness of the plasma TIM-1 contents determined with the developed immunoplatform not only for diagnosis but also for staging of CRC, through the cut-off values shown in Fig. S8. These results are fully consistent with those obtained from solid through pathological anatomy and agree with CRC classification guidelines-TNM classification criteria from the American Joint Committee on Cancer. Therefore, the quantitative plasma contents provided by the developed simple and minimally invasive method in just 60 min are of great interest to add precision to the conventional diagnoses and prognoses, thus contributing to the personalized and more efficient management of patients with CRC.

Additionally, the accuracy of the results obtained with the developed immunoplatform for plasma analysis was evaluated through recovery studies. A representative sample from each group was spiked with 150 pg mL^−1^ of TIM-1 standard. The results obtained using the same protocol as for non-spiked samples and after subtracting the endogenous content from the total concentration measured post-spiking, yielded recovery values between 98 and 104% across the five types of analyzed samples (Table S3, Supporting Information), thus confirming the excellent accuracy of the methodology.

## Conclusions

In this work, the opportunities offered by HOOC-CeO_2_NPs as multifunctional nanolabels in electrochemical immunosensing have been explored. Such NPs function both as nanozymes with robust pseudo-peroxidase activity and stable nanocarriers of detector antibodies. Their use to construct an electrochemical immunosensor involving electrochemical grafting, a sandwich format, and amperometry transduction onto SPCEs, has allowed the sensitive and selective determination of TIM-1, a biomarker of emerging relevance in cancer angiogenesis. The experimental results proved the superior stability of these nanozymes (HOOC-CeO_2_NPs) compared to the natural enzyme (HRP) in the presence of high concentrations of H_2_O_2_, the simplification in the nanolabel preparation (which, unlike previous studies, do not require their combination with other materials or their decoration with HRP molecules), and their ability to improve moderately the sensitivity in electrochemical immunosensing compared to the more conventional enzymatic labeling strategy used in sandwich immunoassays and immunosensors involving the dAb and a HRP-secondary antibody.

The developed dAb/HOOC-CeO_2_NPs-assisted immunoplatform has provided detectability (LOD of 9.9 pg mL^−1^) and a linear concentration range (33–600 pg mL^−1^) compatible with clinical applications and has been successfully applied to the analysis of plasma samples from cancer patients after a simple sample dilution. Interestingly, the results obtained in CRC scenarios demonstrated the potential of determining TIM-1 plasma levels both to diagnose and to stratify this neoplasia, providing the first cut-off values described for this purpose. It is also important to highlight that, according to the state of the art, this was the first electrochemical biotool for TIM-1 that demonstrated applicability in real-world scenarios. The developed immunoplatform was also advantageous over conventional ELISA methodology in terms of applicability in decentralized and resource-limited settings. In addition, the opportunities offered by these multifunctional CeO_2_-based nanomaterials would be transferable and exploitable in other types of biosensing.

Therefore, this work provides both novel and relevant results that highlight the value of this innovative and multifunctional label in immunosensing constituting a good starting point to continue exploiting the potential of this new hybrid nanomaterial with great versatility of modification in affinity biosensing (not only electrochemical). We envision, for example, the development of immunoplatforms involving other formats (for instance, direct competitive configuration by immobilizing the target antigen instead of the detection antibody on CeO_2_NPs) or with in other affinity systems besides immunosensors, such as nucleic acid, peptide or aptameric bioplatforms.

## Supplementary Information

Below is the link to the electronic supplementary material.

Supplementary file1 (DOCX 870 KB)

## Data Availability

The authors confirm that the data supporting the findings of this study are available within the article and its supplementary materials.

## References

[1] Campuzano S, Yáñez-Sedeño P, Pingarrón JM (2020) Electrochemical affinity biosensors based on selected nanostructures for food and environmental monitoring. Sensors 20:5125. 10.3390/s20185125 32911860 10.3390/s20185125 PMC7571223

[2] Kurup CP, Ahmed MU (2023) Nanozymes towards personalized diagnostics: a recent progress in biosensing. Biosensors 13:461. 10.3390/BIOS13040461 37185536 10.3390/bios13040461 PMC10136715

[3] Wang Q, Liu J, He L, Liu S, Yang P (2023) Nanozyme: a rising star for cancer therapy. Nanoscale 15(30):12455–12463. 10.1039/D3NR01976D 37462391 10.1039/d3nr01976d

[4] Diao Q, Chen X, Tang Z, Li S, Tian Q, Bu Z, Liu H, Liu J, Niu X (2024) Nanozymes: powerful catalytic materials for environmental pollutant detection and degradation. Environ Sci: Nano 11:766–796. 10.1039/D3EN00844D

[5] Bilal M, Khaliq N, Ashraf M, Hussain N, Baqar Z, Zdarta J, Jesionowski T, Iqbal HMN (2023) Enzyme mimic nanomaterials as nanozymes with catalytic attributes. Colloids Surf B 221:112950. 10.1016/j.colsurfb.2022.112950 10.1016/j.colsurfb.2022.112950 36327773

[6] Hui W, Erkang W (2013) Nanomaterials with enzyme-like characteristics (nanozymes): next-generation artificial enzymes. Chem Soc Rev 42:6060–6093. 10.1039/C3CS35486E 23740388 10.1039/c3cs35486e

[7] Das B, Franco JL, Logan N, Balasubramanian P, Kim MI, Cao C (2021) Nanozymes in point-of-care diagnosis: an emerging futuristic approach for biosensing. Nano-Micro Lett 13:193. 10.1007/s40820-021-00717-0 10.1007/s40820-021-00717-0 PMC8438099 34515917

[8] Anboo S, Yon Lau S, Kansedo J, Yap P-S, Hadibarata T, Jeevanandam J, Kamaruddin AH (2022) Recent advancements in enzyme-incorporated nanomaterials: synthesis, mechanistic formation, and applications. Biotechnol Bioeng 119:2609–2638. 10.1002/bit.28185 35851660 10.1002/bit.28185 PMC9543334

[9] Li F, Zou L, He J, Wu Y, Yang L, Liu Q, Wu Q, Yang X (2021) On the correlation between structure and catalytic activity of mesoporous ceria nanoparticles. J Catal 402:300–309. 10.1016/j.jcat.2021.08.047

[10] Varvoutis G, Lykaki M, Marnellos GE, Konsolakis MC (2023) Recent advances on fine-tuning engineering strategies of CeO_2_-based nanostructured catalysts exemplified by CO_2_ hydrogenation processes. Catalysts 13:275. 10.3390/catal13020275

[11] Centane S, Nyokong T (2023) Co phthalocyanine mediated electrochemical detection of the HER2 in the presence of Au and CeO_2_ nanoparticles and graphene quantum dots. Bioelectrochemistry 149:108301. 10.1016/j.bioelechem.2022.108301 36272296 10.1016/j.bioelechem.2022.108301

[12] Cheng W, Lin Z, Zhao L, Fan N, Bai H, Cheng W, Zhao M, Ding S (2022) CeO_2_/MXene heterojunction-based ultrasensitive electrochemiluminescence biosensing for BCR-ABL fusion gene detection combined with dual-toehold strand displacement reaction for signal amplification. Biosens Bioelectron 210:114287. 10.1016/j.bios.2022.114287 35500311 10.1016/j.bios.2022.114287

[13] Centane S, Mgidlana S, Openda Y, Nyokong T (2022) Electrochemical detection of human epidermal growth factor receptor 2 using an aptamer on cobalt phthalocyanines – cerium oxide nanoparticle conjugate. Bioelectrochemistry 146:108146. 10.1016/J.BIOELECHEM.2022.108146 35504229 10.1016/j.bioelechem.2022.108146

[14] Pang X, Li J, Zhao Y, Wu D, Zhang Y, Du B, Ma H, Wei Q (2015) Label-Free electrochemiluminescent immunosensor for detection of carcinoembryonic antigen based on nanocomposites of GO/MWCNTs-COOH/Au@CeO_2_. ACS Appl Mater Interfaces 7:19260–19267. 10.1021/ACSAMI.5B05185/ASSET/IMAGES/LARGE/AM-2015-05185Q_0007.JPEG 26271682 10.1021/acsami.5b05185

[15] Pachauri N, Dave K, Dinda A, Solanki PR (2018) Cubic CeO_2_ implanted reduced graphene oxide-based highly sensitive biosensor for non-invasive oral cancer biomarker detection. J Mater Chem B 6:3000–3012. 10.1039/C8TB00653A 32254335 10.1039/c8tb00653a

[16] Wang M, Hu M, Hu B, Guo C, Song Y, Jia Q, He L, Zhang Z, Fang S (2019) Bimetallic cerium and ferric oxides nanoparticles embedded within mesoporous carbon matrix: electrochemical immunosensor for sensitive detection of carbohydrate antigen 19–9. Biosens Bioelectron 135:22–29. 10.1016/J.BIOS.2019.04.018 30991268 10.1016/j.bios.2019.04.018

[17] Yu S, Zou G, Wei Q (2016) Ultrasensitive electrochemical immunosensor for quantitative detection of tumor specific growth factor by using Ag@CeO_2_ nanocomposites as labels. Sens Actuators B Chem 222:314–320. 10.1016/J.SNB.2015.08.117 10.1016/j.talanta.2016.04.050 27260429

[18] Li Y, Zhang Y, Li F, Feng J, Li M, Chen L, Dong Y (2017) Ultrasensitive electrochemical immunosensor for quantitative detection of SCCA using Co_3_O_4_@CeO_2_-Au@Pt nanocomposite as enzyme-mimetic labels. Biosens Bioelectron 92:33–39. 10.1016/j.bios.2017.01.065 28182976 10.1016/j.bios.2017.01.065

[19] Ramos-López C, García-Rodrigo L, Sánchez-Tirado E, Agüí L, González-Cortés A, Yáñez-Sedeño P, Pingarrón JM (2024) Cerium dioxide-based nanostructures as signal nanolabels for current detection in the immunosensing determination of salivary myeloperoxidase. Microchem J 201:110505. 10.1016/J.MICROC.2024.110505

[20] Quinchia J, Blázquez-García M, Torrente-Rodríguez RM, Ruiz-Valdepeñas Montiel V, Serafín V, Rejas-González R, Montero-Calle A, Orozco J, Pingarrón JM, Barderas R, Campuzano S (2024) Disposable electrochemical immunoplatform to shed light on the role of the multifunctional glycoprotein TIM-1 in cancer cells invasion. Talanta 267:125155. 10.1016/J.TALANTA.2023.125155 37696234 10.1016/j.talanta.2023.125155

[21] Lee SH, Lee HS, Park G, Oh SM, Oh DS (2019) Dual actions on gout flare and acute kidney injury along with enhanced renal transporter activities by Yokuininto, a Kampo Medicine. BMC Compl Altern Med 19:57. 10.1186/s12906-019-2469-9 10.1186/s12906-019-2469-9 PMC6419507 30871515

[22] Vaidya VS, Ford GM, Waikar SS, Wang Y, Clement MB, Ramirez V, Glaab WE, Troth SP, Sistare FD, Prozialeck WC, Edwards JR, Bobadilla NA, Mefferd SC, Bonventre JV (2009) A rapid urine test for early detection of kidney injury. Kidney Int 76(1):108–114. 10.1038/ki.2009.96 19387469 10.1038/ki.2009.96 PMC2737345

[23] Chung HJ, Pellegrini KL, Chung J, Wanigasuriya K, Jayawardene I, Lee K, Lee H, Vaidya VS, Weissleder R (2015) Nanoparticle detection of urinary markers for point-of-care diagnosis of kidney injury. PLoS One 10(7):e0133417. 10.1371/journal.pone.0133417 26186708 10.1371/journal.pone.0133417 PMC4506142

[24] Yang H, Wang H, Xiong C, Chai Y, Yuan R (2018) Highly sensitive electrochemiluminescence immunosensor based on ABEI/H_2_O_2_ system with PFO dots as enhancer for detection of kidney injury molecule-1. Biosens Bioelectron 116:16–22. 10.1016/J.BIOS.2018.05.032 29852472 10.1016/j.bios.2018.05.032

[25] Liu C, Yang W, Min X, Zhang D, Fu X, Ding S, Xu W (2021) An enzyme-free electrochemical immunosensor based on quaternary metallic/nonmetallic PdPtBP alloy mesoporous nanoparticles/MXene and conductive CuCl_2_ nanowires for ultrasensitive assay of kidney injury molecule-1. Sens Actuators B Chem 334:129585. 10.1016/J.SNB.2021.129585

[26] Boyacıoğlu H, Yola BB, Karaman C, Karaman O, Atar N, Yola ML (2022) A novel electrochemical kidney injury molecule-1 (KIM-1) immunosensor based covalent organic frameworks-gold nanoparticles composite and porous NiCo_2_S_4_@CeO_2_ microspheres: the monitoring of acute kidney injury. Appl Surf Sci 578:152093. 10.1016/j.apsusc.2021.152093

[27] Das M, Chakraborty T, Lin CY, Lei KF, Kao CH (2023) Hierarchical gold-Galinstan nanodendrites modified disposable immunosensor for the label-free detection of KIM-1 by antibody immobilization on staphylococcal protein A. Appl Surf Sci 607:154930. 10.1016/J.APSUSC.2022.154930

[28] Chen HI, Chang HY (2005) Synthesis of nanocrystalline cerium oxide particles by the precipitation method. Ceram Int 31:795–802. 10.1016/J.CERAMINT.2004.09.006

[29] Such Basáñez I (2015) Inmovilización de complejos organometálicos en soportes sólidos para aplicación en catálisis. Tesis doctoral, Universidad de Alicante. http://rua.ua.es/dspace/handle/10045/47349. Accessed 26 Nov 2024

[30] Farahmandjou M, Zarinkamar M, Firoozabadi TP (2016) Synthesis of cerium oxide (CeO_2_) nanoparticles using simple CO-precipitation method. Rev Mex Fis 62:496–499. http://www.scielo.org.mx/scielo.php?script=sci_arttext&pid=S0035-001X2016000500496&lng=es&nrm=iso&tlng=en. Accessed 26 Nov 2024

[31] The International Centre for Diffraction Data - ICDD. https://www.icdd.com/. Accessed 18 October 2024

[32] Estevez AY, Ganesana M, Trentini JF, Olson JE, Li G, Boateng YO, Lipps JM, Yablonski SER, Donnelly WT, Leiter JC, Erlichman JS (2019) Antioxidant enzyme-mimetic activity and neuroprotective effects of cerium oxide nanoparticles stabilized with various ratios of citric acid and EDTA. Biomolecules 9:562. 10.3390/BIOM9100562 31623336 10.3390/biom9100562 PMC6843313

[33] Patil S, Sandberg A, Heckert E, Self W, Seal S (2007) Protein adsorption and cellular uptake of cerium oxide nanoparticles as a function of zeta potential. Biomaterials 28:4600–4607. 10.1016/J.BIOMATERIALS.2007.07.029 17675227 10.1016/j.biomaterials.2007.07.029 PMC2259388

[34] Huang B, Yao C, Yang J, Dua S, Lu X (2020) A study on the electrochemical behavior of hydroquinone at a nanometer cobalt/L-glutamate modified electrode. RSC Adv 10:43834–43839. 10.1039/D0RA07222B 35519711 10.1039/d0ra07222b PMC9058242

[35] Kushlinskii NE, Gershtein ES, Naberezhnov DS, Taipov MA, Bezhanova SD, Pushkar’ DY, Matveev VB, Stilidi IS (2019) Kidney Injury Molecule-1 (KIM-1) in blood plasma of patients with clear-cell carcinoma. Bull Exp Biol Med 167:388–392. 10.1007/S10517-019-04533-W 31346876 10.1007/s10517-019-04533-w

[36] Ben QW, Zhao Z, Ge SF, Zhou J, Yuan F, Yuan YZ (2009) Circulating levels of periostin may help identify patients with more aggressive colorectal cancer. Int J Oncol 34:821–828. 10.3892/ijo_00000208 19212687 10.3892/ijo_00000208

[37] Barderas R, Bartolomé RA, Fernández-Aceñero MJ, Torres S, Casal JI (2012) High expression of IL-13 receptor α2 in colorectal cancer is associated with invasion, liver metastasis, and poor prognosis. Cancer Res 72:2780–2790. 10.1158/0008-5472.CAN-11-4090 22505647 10.1158/0008-5472.CAN-11-4090

[38] Al Obeed OA, Alkhayal KA, Al Sheikh A, Zubaidi AM, Vaali-Mohammed MA, Boushey R, Mckerrow JH, Abdulla MH (2014) Increased expression of tumor necrosis factor-α is associated with advanced colorectal cancer stages. World J Gastroenterol 20:18390–18396. 10.3748/wjg.v20.i48.18390 25561807 10.3748/wjg.v20.i48.18390 PMC4277977

[39] Li Z, Zhang X, Yang Y, Yang S, Dong Z, Du L, Wang L, Wang C (2015) Periostin expression and its prognostic value for colorectal cancer. Int J Mol Sci 16:12108. 10.3390/IJMS160612108 26023718 10.3390/ijms160612108 PMC4490432

[40] Deng X, Ao S, Hou J, Li Z, Lei Y, Lyu G (2019) Prognostic significance of periostin in colorectal cancer. Chin J Cancer Res 31:547. 10.21147/J.ISSN.1000-9604.2019.03.16 31354223 10.21147/j.issn.1000-9604.2019.03.16 PMC6613499

[41] Warsinggih LF, Labeda I, Lusikooy RE, Mappincara Faruk M (2021) The relationship of tumor necrosis factor alpha levels in plasma toward the stage and differentiation degree in colorectal cancer. Med Clín Práct 4:100224. 10.1016/J.MCPSP.2021.100224

