# Correction: Multifunctional cerium nanolabels in electrochemical immunosensing with improved robustness and performance: determination of TIM-1 in colorectal cancer scenarios as a case study

**DOI:** 10.1007/s00604-025-07561-8

**Published:** 2025-09-23

**Authors:** Andrea Cabrero-Martín, Sara Santiago, Verónica Serafín, María Pedrero, Ana Montero-Calle, José M. Pingarrón, Rodrigo Barderas, Susana Campuzano

**Affiliations:** 1 Departamento de Química Analítica, Facultad de CC. Químicas, Universidad Complutense de Madrid, Pza. de Las Ciencias 2, 28040 Madrid, Spain; 2 Chronic Disease Programme, UFIEC, Institute of Health Carlos III, Majadahonda, 28220 Madrid, Spain; 3 CIBER of Frailty and Healthy Aging (CIBERFES), Instituto de Salud Carlos III, 28046 Madrid, Spain

**Correction: Microchimica Acta (2025) 192:243**

10.1007/s00604-025-07021-3

The article "Multifunctional cerium nanolabels in electrochemical immunosensing with improved robustness and performance: determination of TIM-1 in colorectal cancer scenarios as a case study", written by Andrea Cabrero-Martín, Sara Santiago, Verónica Serafín, María Pedrero, Ana Montero-Calle, José M. Pingarrón, Rodrigo Barderas & Susana Campuzano, was originally published under exclusive license to The Author(s), under exclusive licence to Springer-Verlag GmbH Austria, part of Springer Nature. As a result of the subsequent decision to publish the article under the open access model, the article’s copyright notice was changed on 28 August 2025 to © The Author(s) 2025 and the article is now distributed under a Creative Commons Attribution 4.0 International License, which permits use, sharing, adaptation, distribution and reproduction in any medium or format, as long as you give appropriate credit to the original author(s) and the source, provide a link to the Creative Commons licence, and indicate if changes were made.

The images or other third party material in this article are included in the article’s Creative Commons licence, unless indicated otherwise in a credit line to the material. If material is not included in the article’s Creative Commons licence and your intended use is not permitted by statutory regulation or exceeds the permitted use, you will need to obtain permission directly from the copyright holder.

To view a copy of this licence, visit http://creativecommons.org/licenses/by/4.0.

