# Supplementary material for: Multifunctional cerium nanolabels in electrochemical immunosensing with improved robustness and performance: determination of TIM-1 in colorectal cancer scenarios as a case study
Source: Mikrochim Acta. 2025 Mar 19;192(4):243. doi: 10.1007/s00604-025-07021-3 (PMC12443898; doi:10.1007/s00604-025-07021-3)
Supplement: Supplementary file 1 — Supplementary file1 (DOCX 870 KB) [file 604_2025_7021_MOESM1_ESM.docx]

SUPPORTING INFORMATION (SI)

**Multifunctional Cerium Nanolabels in Electrochemical Immunosensing with Improved Robustness and Performance. Determination of TIM-1 in Colorectal Cancer Scenarios as a Case Study**

Andrea Cabrero-Martín^1‡^, Sara Santiago^1‡^, Verónica Serafín^1^, María Pedrero^1^, Ana Montero-Calle^2^, José M. Pingarrón^1^, Rodrigo Barderas^2,3^*, Susana Campuzano^1,3^*

^‡^These authors contributed equally to this work and shared first authorship

^1^Departamento de Química Analítica, Facultad de CC. Químicas, Universidad Complutense de Madrid, Pza. de las Ciencias 2, Madrid 28040, Spain.

^2^Chronic Disease Programme, UFIEC, Institute of Health Carlos III, Majadahonda, Madrid 28220, Spain.

^3^CIBER of Frailty and Healthy Aging (CIBERFES), Instituto de Salud Carlos III, Madrid, 28046 Spain

E-mail: [susanacr@quim.ucm.es](mailto:susanacr@quim.ucm.es); E-mail: [r.barderasm@isciii.es](mailto:r.barderasm@isciii.es)

| **Contents** | **Page/s** |
| --- | --- |
| **Fig. S1** | S2 |
| **Fig. S2** | S2 |
| **Table S1** | S2 |
| **Fig. S3**  **Fig. S4** | S3  S4 |
| **Table S2** | S6 |
| **Fig. S5** | S6 |
| **Fig. S6** | S7 |
| **Fig. S7** | S7 |
| **Fig. S8** | S8 |
| **Table S3** | S8 |
| **References** | S8 |


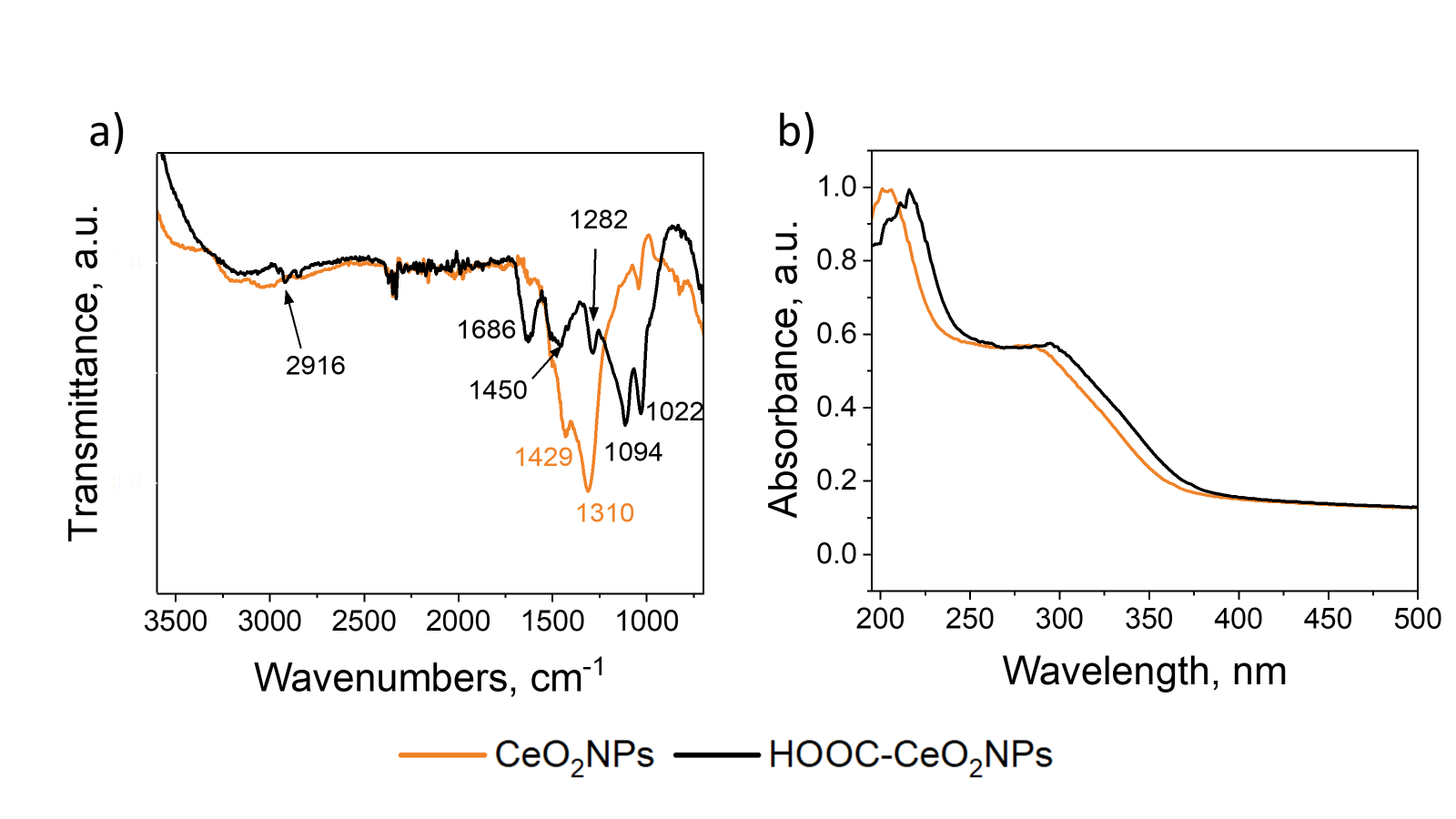


**Fig. S1** a) FTIR and b) UV-Vis spectra of CeO_2_ NPs (orange line) and HOOC-CeO_2_ NPs (black line).


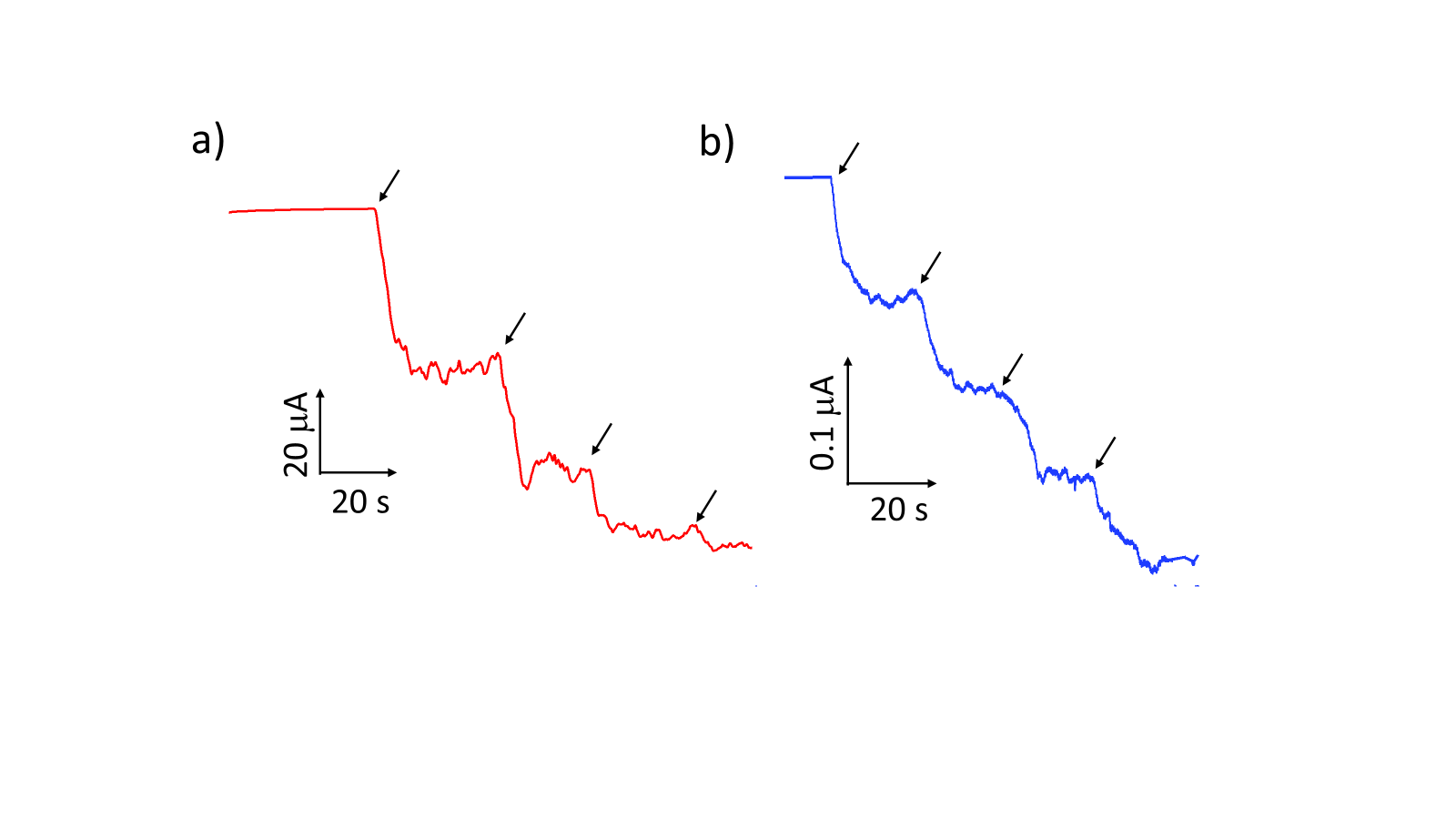


**Fig. S2** Comparison of amperometric responses obtained with HRP/SPCE and dAb/HOOC-CeO_2_NPs/SPCE for 50 μL additions of 0.3 M H_2_O_2_ to an electrochemical cell containing 10 mL of 50 mM PB at pH 6.0 supplemented with 50 μL of 100 mM HQ solution.

**Table S1** Apparent rate constants calculated for the tested enzyme/nanozymes.

| **Enzyme/Nanozyme** | **K_app_, min^−1^** | **R^2^** |
| --- | --- | --- |
| HRP | 5.43 ± 0.03 | 0.998 |
| CeO_2_NPs | 1.68 ± 0.01 | 0.999 |
| HOOC-CeO_2_NPs | 1.40 ± 0.09 | 0.998 |
| dAb/HOOC-CeO_2_NPs | 1.54 ± 0.01 | 0.999 |


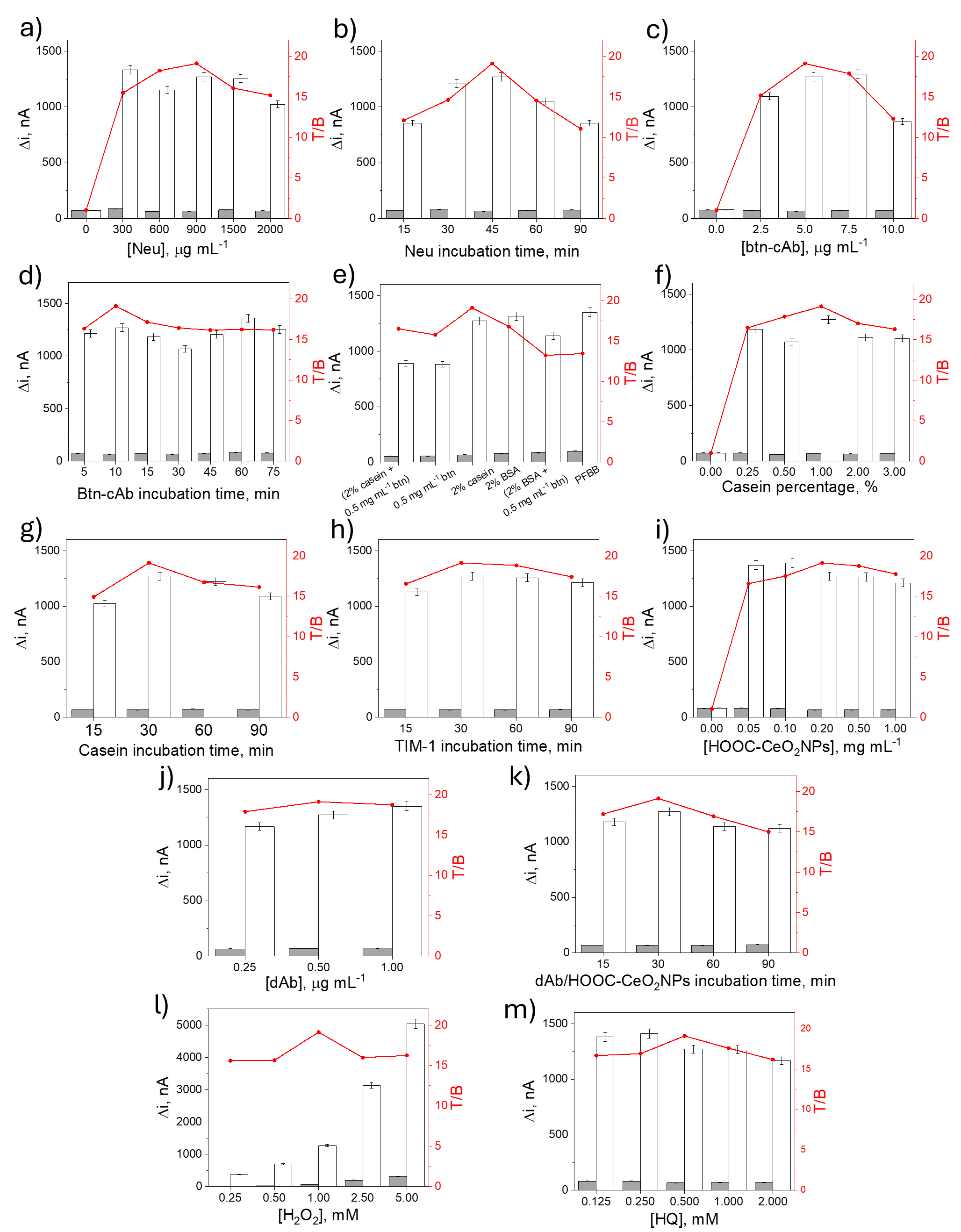


**Fig. S3** Amperometric responses measured in the presence (blue bars, T signals) and absence (grey bars, B signals) of 500 pg mL⁻¹ TIM-1 standard, along with the corresponding T/B ratio values (in red), as a function of: a) Neu concentration; b) Neu incubation time; c) btn-cAb concentration; d) btn-cAb incubation time; e) blocking solution; f) casein percentage; g) casein incubation time; h) TIM-1 incubation time; i) HOOC-CeO_2_NPs loading; j) dAb loading; k) dAb/HOOC-CeO_2_NPs incubation time; l) H_2_O_2_ concentration; m) HQ concentration.

**
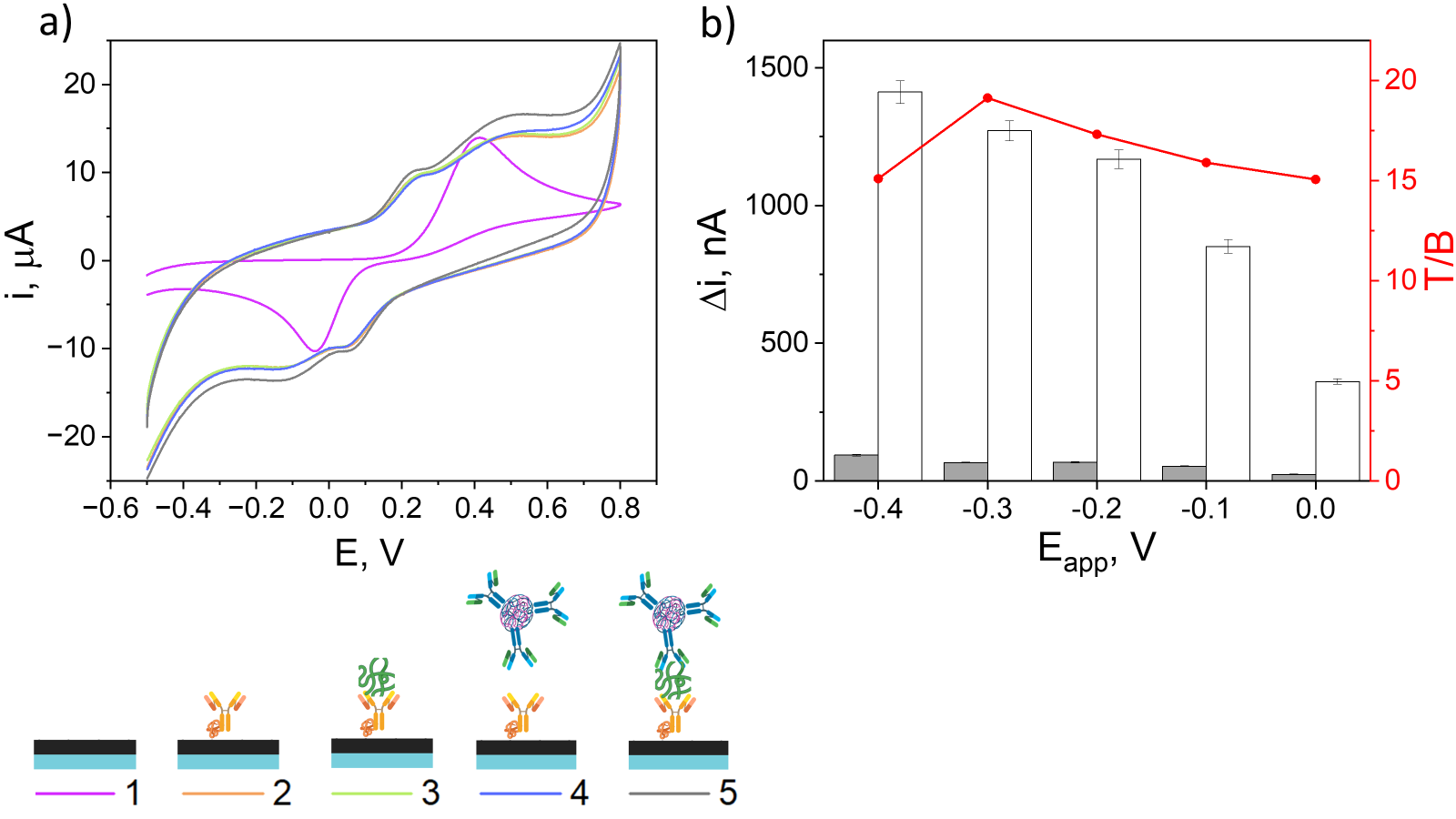
**

**Fig. S4** a) Cyclic voltammograms (scan rate, 50 mV s⁻¹) obtained for 0.5 mM HQ in the presence of 1.0 mM H_2_O_2_ at the bare SPCE (purple), btn-cAb/SPCE (orange), TIM-1/btn-cAb/SPCE (green), dAb/HOOC-CeO_2_NPs/btn-cAb/SPCE (blue) and dAb/HOOC-CeO_2_NPs/TIM-1/btn-cAb/SPCE (grey). b) Amperometric responses measured in the presence (white bars, T signals) and absence (grey bars, B signals) of 500 pg mL⁻¹ TIM-1 standard, along with the corresponding T/B ratio values (in red), as a function of the applied detection potential.

**Table S2** Optimization of experimental variables involved in the preparation and operation of the developed immunoplatform.

| **Variable** | **Tested Range** | **Selected Value** |
| --- | --- | --- |
| [Neu], μg mL^−1^ | 0 – 2000.0 | 900.0 |
| Neu incubation time, min | 15 – 90 | 45 |
| [Btn-cAb], μg mL^−1^ | 0.0 – 10.0 | 5.0 |
| Btn-cAb incubation time, min | 5 – 75 | 10 |
| Blocking mixture* | A – F | B |
| [Casein], % (wt.) | 0.2 – 3.0 | 1.0 |
| Casein incubation time, min | 15 – 90 | 30 |
| TIM-1 incubation time, min | 15 – 90 | 30 |
| [HOOC-CeO_2_NPs], mg mL^−1^ | 0.0 – 1.0 | 0.2 |
| [dAb], µg mL^−1^ | 0.25 – 5.0 | 0.5 |
| dAb/HOOC-CeO_2_NPs incubation time, min | 15 – 90 | 30 |
| [H_2_O_2_], mM | 0.25 – 5.0 | 1.0 |
| [HQ], mM | 0.125 – 2.0 | 0.5 |
| E_app_, V** | (− 0.4) – (0.0) | − 0.2 |

** (A) 0.5 mg mL^−1^ btn, (B) 2 % casein, (C) 2 % casein + 0.5 mg mL^−1^ btn, (D) 2 % BSA, (E) 2 % BSA + 0.5 mg mL^−1^ btn, (F) PFBB. ** vs. Ag pseudo-reference electrode.*

*
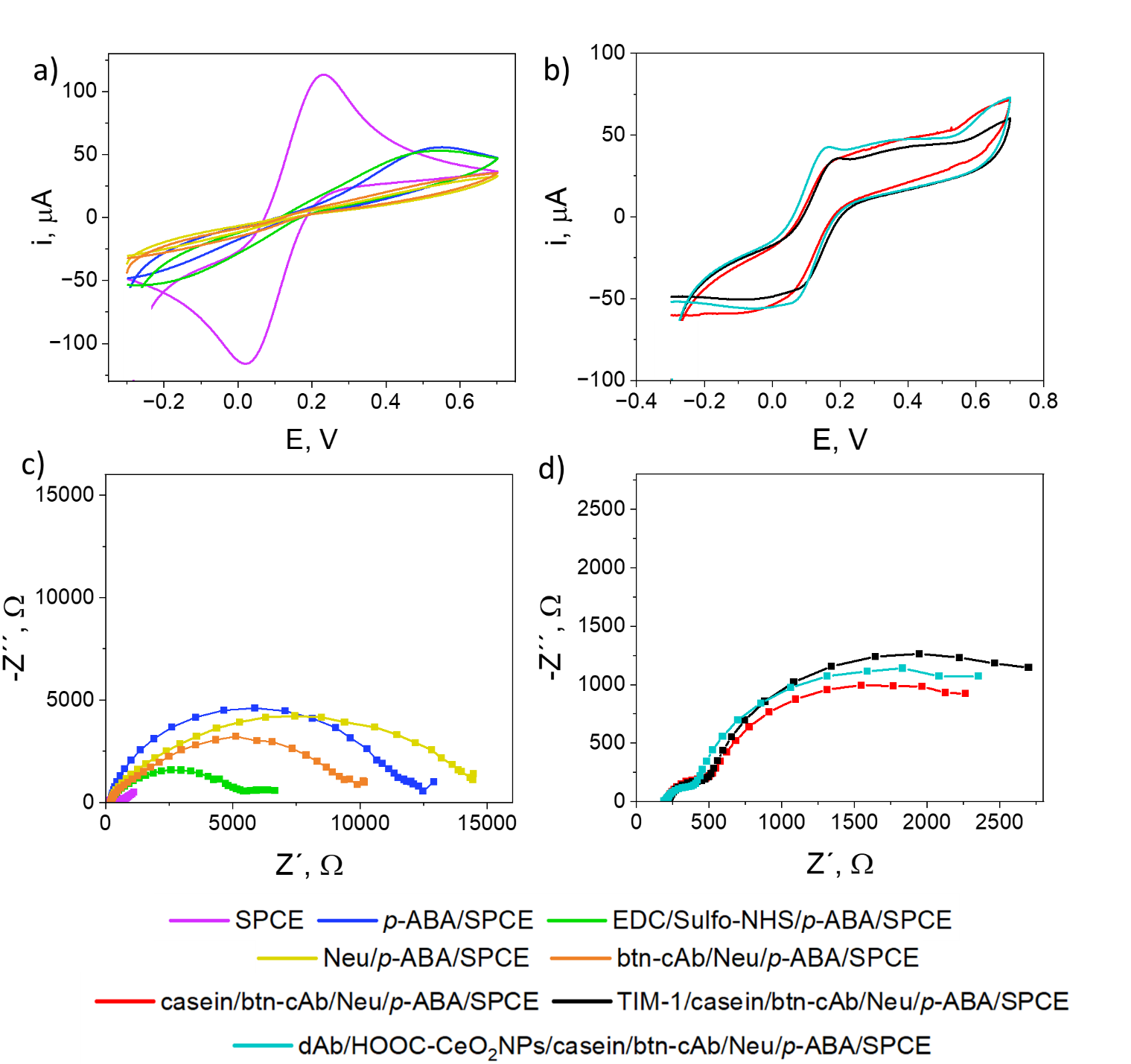
*

**Fig. S5** a, b) Nyquist plots for the [Fe(CN)₆]^3‒/4‒^ redox pair recorded after each step involved in the immunoplatform preparation (n° of frequencies = 50; frequency sweep: 10^5^ – 0.04 Hz; amplitude (rms) = 0.01 V, fitted to the equivalent circuits R1(Q1[R2W1]) a) or R1(R2Q1)(Q2[R3W1]) b). c, d) Cyclic voltammograms of the [Fe(CN)₆]^3‒/4‒^ redox pair (potential scan sweep: − 0.3 V/+ 0.7 V; scan rate: 50 mV s⁻¹). SPCE (purple); *p*-ABA/SPCE (dark blue); EDC/sulfo-NHS/*p*-ABA/SPCE, (green); Neu/*p*-ABA/SPCE (yellow); btn-cAb/Neu/*p*-ABA/SPCE (orange); casein/btn-cAb/Neu/*p*-ABA/SPCE (red); TIM-1/casein/btn-cAb/Neu/*p*-ABA/SPCE (black); and dAb/HOOC-CeO_2_NPs/TIM-1/casein/btn-cAb/Neu/*p*-ABA/SPCE (light blue).


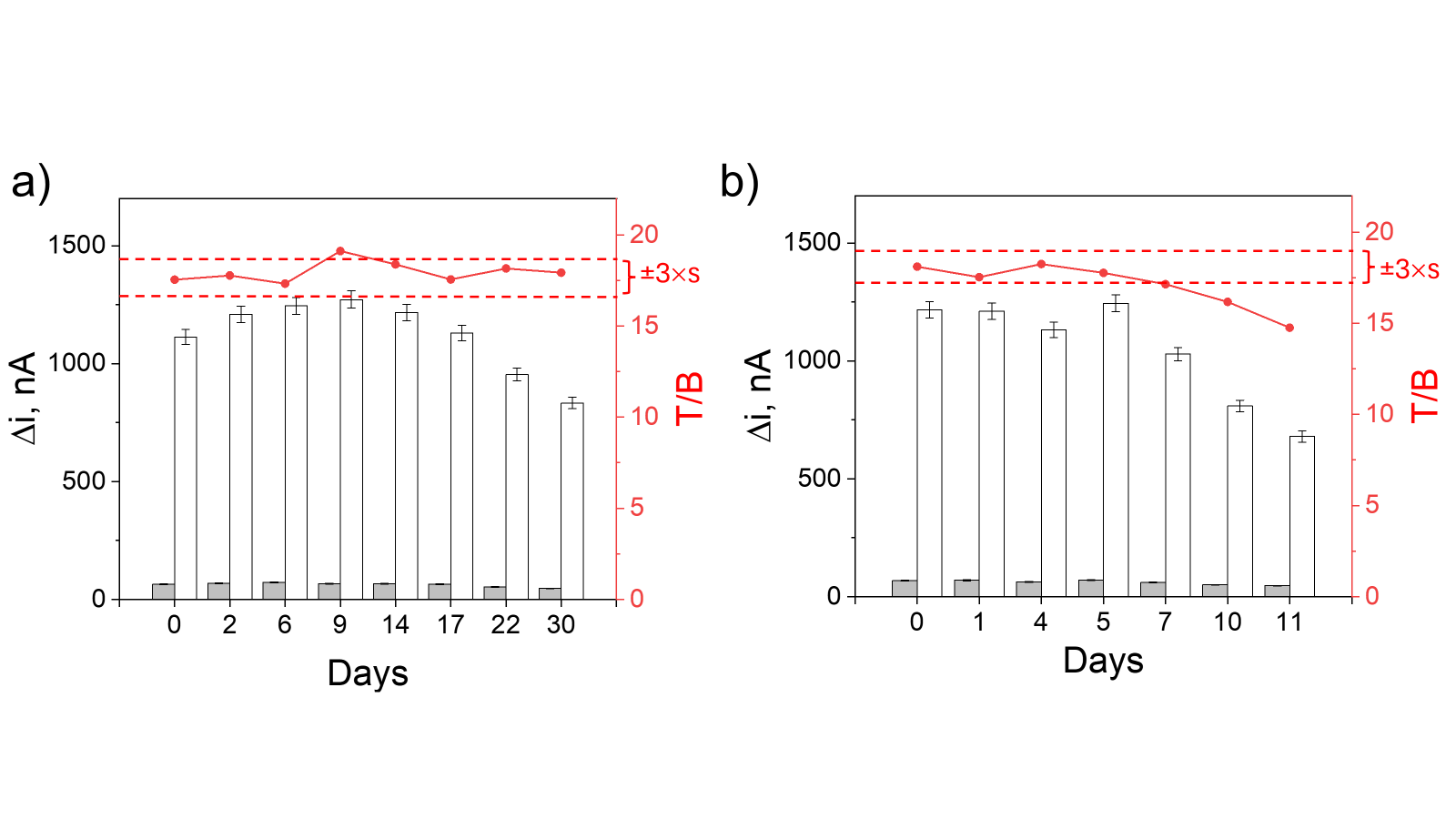


**Fig. S6** Storage stability of a) the immunoplatform (casein/btn-cAb/Neu/p-ABA-SPCEs, at 4 °C in a humid chamber) and b) the dAb/HOOC-CeO₂NPs (stored in PBST at 4 °C under constant stirring). Amperometric responses were obtained in the absence (grey bars) and in the presence (white bars) of 500 pg mL⁻¹ TIM-1, along with the corresponding T/B ratio values (red dots and lines). Control limits were defined as ± 3 times the standard deviation of three measurements performed on the day of preparation (day 0).

**
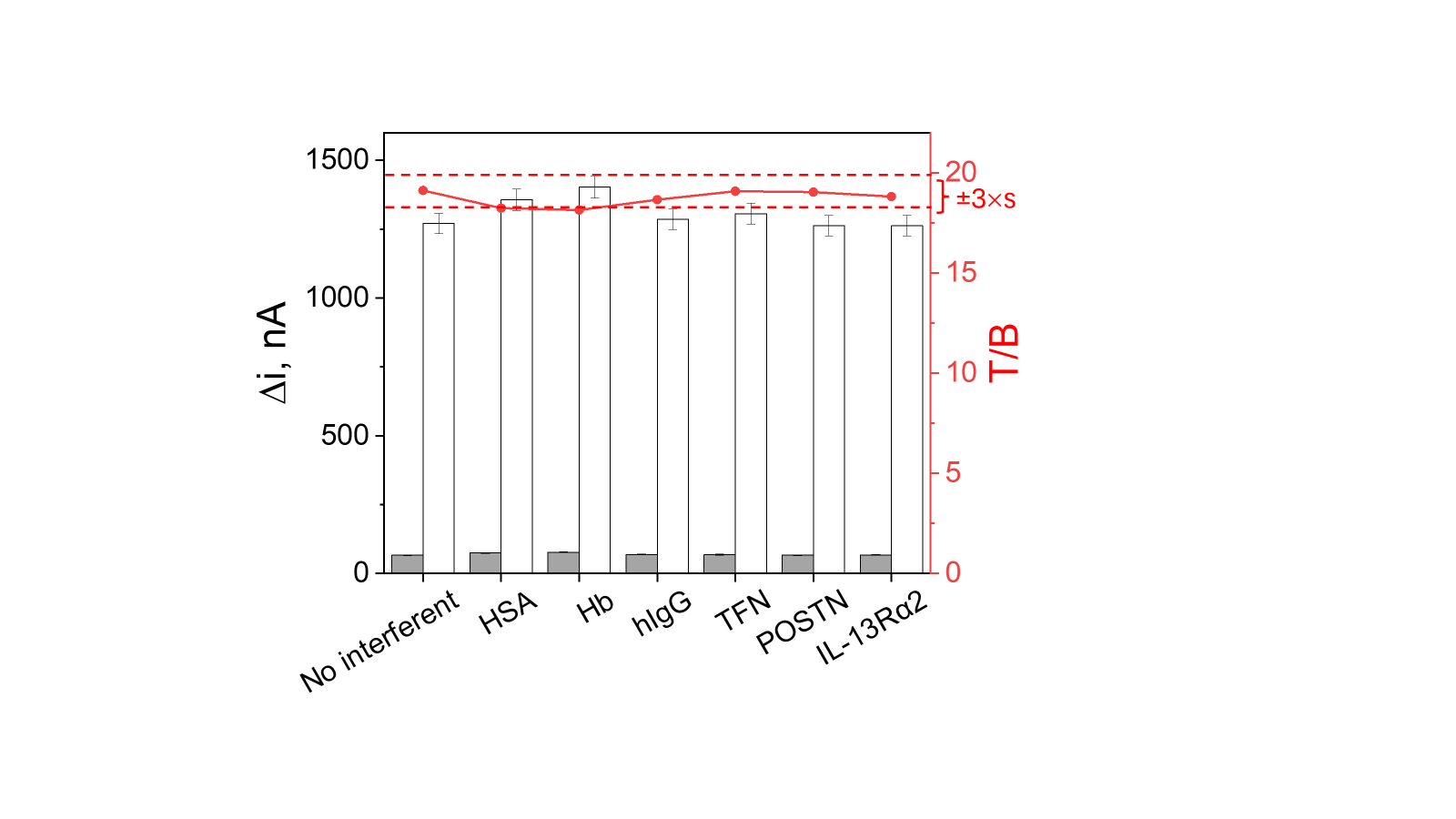
**

**Fig. S7** Amperometric responses obtained with the immunoplatforms for 0 (gray bars) and 500 pg mL⁻¹ (white bars) TIM-1 prepared in the absence (“No interferent”) and in the presence of 1 mg mL⁻¹ hIgG, 50 mg mL⁻¹ HSA, 2.5 mg mL⁻¹ Hb, 5 ng mL⁻¹ TNF, 5 ng mL⁻¹ POSTN, and 50 ng mL⁻¹ IL-13Rα2.

**
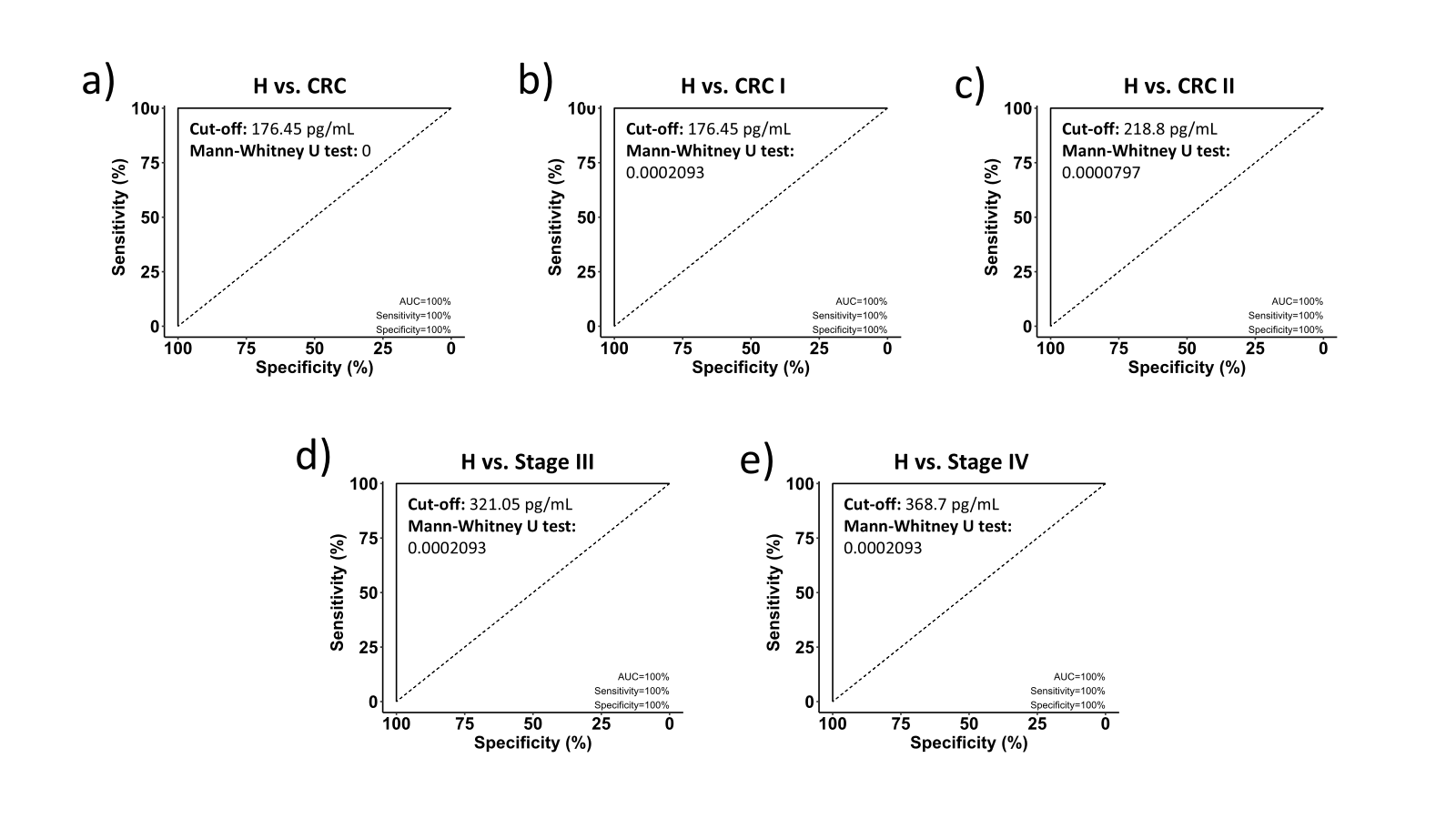
**

**Fig. S8** Analysis of the quantitative results obtained with the developed immunoplatform for the determination of TIM-1by means of ROC curves.

**Table S3** TIM-1 recoveries in plasma samples from healthy (H) and colorectal (CRC) patients at different stages.

| Sample | % Recovery |
| --- | --- |
| H | 98.2 |
| stage I CRC | 99.2 |
| stage II CRC | 104.0 |
| stage III CRC | 100.6 |
| stage IV CRC | 100.3 |

References

S1. Wang Y, Martin TA, Jiang WG (2013) HAVcR-1 expression in human colorectal cancer and its effects on colorectal cancer cells in vitro. Anticancer Res 33(1):207–214. PMID: 2326S57147

S2. Telford EJA, Jiang WG, Martin TA (2017) HAVcR-1 involvement in cancer progression. Histopathology 32:121–128. <https://doi.org/10.14670/HH-11-817>

S3. Zheng X, Xu K, Chen L, Zhou Y, Jiang J (2019) Prognostic value of TIM-1 expression in human non-small-cell lung cancer. J Transl Med 17:178. <https://doi.org/10.1186/s12967-019-1931-2>

S4. Kong X, Fu M, Niu X, Jiang H (2020) Comprehensive analysis of the expression, relationship to immune infiltration and prognosis of TIM-1 in cancer. Front Oncol 10:1086. <https://doi.org/10.3389/fonc.2020.01086>

S5. Chen L, Qing J, Xiao Y, Huang X, Chi Y, Chen Z (2022) TIM-1 promotes proliferation and metastasis, and inhibits apoptosis, in cervical cancer through the PI3K/AKT/P53 pathway. BMC Cancer 22:370. <https://doi.org/10.1186/s12885-022-09386-7>

S6. Wichukchinda N, Nakajima T, Saipradit N, Nakayama EE, Ohtani H, Rojanawiwat A, Pathipvanich P, Ariyoshi K, Sawanpanyalert P, Shioda T, Kimura A (2010) TIM1 haplotype may control the disease progression to AIDS in a HIV-1-infected female cohort in Thailand. AIDS 24:1625–1631. <https://doi.org/10.1097/QAD.0b013e32833a8e6d>

S7. Mosbruger TL, Duggal P, Goedert JJ, Kirk GD, Hoots WK, Tobler LH, Busch M, Peters MG, Rosen HR, Thomas DL, Thio C (2010) Large-scale candidate gene analysis of spontaneous clearance of hepatitis C virus. J Infect Dis 201:1371–1380. <https://doi.org/10.1086/651606>

S8. Kim HY, Eyheramonho MB, Pichavant M, Cambaceres CG, Matangkasombut P, Cervio G, Kuperman S, Moreiro R, Konduru K, Manangeeswaran M, Freeman GJ (2011) A polymorphism in TIM1 is associated with susceptibility to severe hepatitis A virus infection in humans. The Journal of Clinical Investigation 121:1111–1118. <https://doi.org/10.1172/JCI44182>

S9. Hu S, Xie Y, Zhou N, Jin L, Tan Y, Liu D, Gong Y, Liu L, Liu J, Liu W, Chen Y (2011) Expression of T-cell immunoglobulin-and mucin-domain-containing molecules-1 and-3 (Tim-1 and Tim-3) in Helicobacter pylori infection. Helicobacter 16:373–381. <https://doi.org/10.1111/j.1523-5378.2011.00855.x>

S10. Duanghathaipornsuk S, Alateeq FAO, Kim SS, Kim DS, Alba-Rubio AC (2020) The effects of size and content of cerium oxide nanoparticles on a composite sensor for hydroxyl radicals detection. Sens Actuators B 321:128467. <https://doi.org/10.1016/J.SNB.2020.128467>

S11. Serafín V, Valverde A, Martínez-García G, Martínez-Periñán E, Comba F, Garranzo-Asensio M, Barderas R, Yáñez-Sedeño P, Campuzano S, Pingarrón JM (2019) Graphene quantum dots-functionalized multi-walled carbon nanotubes as nanocarriers in electrochemical immunosensing. Determination of IL-13 receptor α2 in colorectal cells and tumor tissues with different metastatic potential. Sens Actuators B Chem 284:711–722. <https://doi.org/10.1016/J.SNB.2019.01.012>

S12. Sánchez-Tirado E, González-Cortés A, Yáñez-Sedeño P, Pingarrón JM (2018) Magnetic multiwalled carbon nanotubes as nanocarrier tags for sensitive determination of fetuin in saliva. Biosens Bioelectron, 113:88–94. <https://doi.org/10.1016/J.BIOS.2018.04.056>

S13. Martínez-García G, Agüí L, Yáñez-Sedeño P, Pingarrón JM (2016) Multiplexed electrochemical immunosensing of obesity-related hormones at grafted graphene-modified electrodes. Electrochim Acta 202:209–215. <https://doi.org/10.1016/J.ELECTACTA.2016.03.140>

S14. Arévalo B, Blázquez-García M, Valverde A, Serafín V, Montero-Calle A, Solís-Fernández G, Barderas R, Campuzano S, Yáñez-Sedeño P, Pingarrón JM (2022) Binary MoS_2_ nanostructures as nanocarriers for amplification in multiplexed electrochemical immunosensing: Simultaneous determination of B cell activation factor and proliferation-induced signal immunity-related cytokines. Microchim Acta 189:143. <https://doi.org/10.1007/s00604-022-05250-4>.

S15. Sigma-Aldrich. Enzymatic assay of peroxidase (EC 1.11.1.7) 2,2’-Azino-bis(3-Ethylbenzthiazoline-6-Sulfonic Acid) as a substrate. <https://www.sigmaaldrich.com/ES/es/technical-documents/protocol/protein-biology/enzyme-activity-assays/enzymatic-assay-of-peroxidase-abts-as-substrate>. Accesed 4 August 2024

S16. Liu YH, Zuo JC, Ren XF, Yong L (2014) Synthesis and character of cerium oxide (CeO₂) nanoparticles by the precipitation method. Metalurgia 53:463–465. <https://hrcak.srce.hr/file/180642>

S17. Latha P, Prakash K, Karuthapandian S (2018) Effective photodegradation of CR & MO dyes by morphologically controlled cerium oxide nanocubes under visible light illumination. Optik 154:242–250. <https://doi.org/10.1016/J.IJLEO.2017.10.054>

S18. Tao Y, Wang H, Xia Y, Zhang G, Wu H, Tao G (2010) Preparation of shape-controlled CeO₂ nanocrystals via microwave-assisted method. Mater Chem Phys 124:541–546. <https://doi.org/10.1016/J.MATCHEMPHYS.2010.07.007>

S19. Kaushik A, Solanki PR, Ansari AA, Ahmad S, Malhotra BD (2009) A nanostructured cerium oxide film-based immunosensor for mycotoxin detection. Nanotechnology 20:055105. <https://doi.org/10.1088/0957-4484/20/5/055105>

S20. Campuzano S. (2004) Desarrollo de biosensores amperométricos enzimáticos basados en monocapas autoensambladas. Tesis doctoral, Universidad Complutense de Madrid. <https://dialnet.unirioja.es/servlet/tesis?codigo=195611&info=resumen&idioma=SPA>

S21. Serafín V (2015) Biosensores electroquímicos basados en nanomateriales y en materiales magnéticos para la determinación de analitos de interés bioquímico. Tesis doctoral, Universidad Complutense de Madrid. <https://hdl.handle.net/20.500.14352/26206>

S22. Cao-Milán R, He LD, Shorkey S, Tonga GY, Wang LS, Zhang X, Uddin I, Das R, Sulak M, Rotello VM (2017) Modulating the catalytic activity of enzyme-like nanoparticles through their surface functionalization. Mol Syst Des Eng 2:624–628. <https://doi.org/10.1039/C7ME00055C>

S23. Morales-Urrea D, López-Córdoba A, Contreras EM (2023) Inactivation kinetics of horseradish peroxidase (HRP) by hydrogen peroxide. Sci Rep 13:13363. <https://doi.org/10.1038/s41598-023-39687-1>

S24. Thermo Fisher Scientific. Avidin and streptavidin conjugates. <https://www.thermofisher.com/es/es/home/references/molecular-probes-the-handbook/antibodies-avidins-lectins-and-related-products/avidin-streptavidin-neutravidin-and-captavidin-biotin-binding-proteins-and-affinity-matrices.html>. Accessed 20 November 2024

S25. [Valverde](https://chemistry-europe.onlinelibrary.wiley.com/authored-by/Valverde/Alejandro) A, [Serafín](https://chemistry-europe.onlinelibrary.wiley.com/authored-by/Seraf%C3%ADn/Ver%C3%B3nica) V, [Montero-Calle](https://chemistry-europe.onlinelibrary.wiley.com/authored-by/Montero%E2%80%90Calle/Ana) A, [González-Cortés](https://chemistry-europe.onlinelibrary.wiley.com/authored-by/Gonz%C3%A1lez%E2%80%90Cort%C3%A9s/Araceli) A, [Barderas](https://chemistry-europe.onlinelibrary.wiley.com/authored-by/Barderas/Rodrigo) R, [Yáñez-Sedeño](https://chemistry-europe.onlinelibrary.wiley.com/authored-by/Y%C3%A1%C3%B1ez%E2%80%90Sede%C3%B1o/Paloma) P, [Campuzano](https://chemistry-europe.onlinelibrary.wiley.com/authored-by/Campuzano/Susana) S, [Pingarrón](https://chemistry-europe.onlinelibrary.wiley.com/authored-by/Pingarr%C3%B3n/Jos%C3%A9+M.) JM (2020) Carbon/inorganic hybrid nanoarchitectures as carriers for signaling elements in electrochemical immunosensors: First biosensor for the determination of the inflammatory and metastatic processes biomarker RANK-ligand. ChemElectroChem 7:810. <https://doi.org/10.1002/celc.201902025>

.
